# Determination of the boundary lipids of sticholysins using tryptophan quenching

**DOI:** 10.1038/s41598-022-21750-y

**Published:** 2022-10-15

**Authors:** Juan Palacios-Ortega, Rafael Amigot-Sánchez, Carmen García-Montoya, Ana Gorše, Diego Heras-Márquez, Sara García-Linares, Álvaro Martínez-del-Pozo, J. Peter Slotte

**Affiliations:** 1 Biochemistry Department, Faculty of Science and Engineering, Åbo Akademi University, Turku, Finland; 2 Departamento de Bioquímica y Biología Molecular, Universidad Complutense, Madrid, Spain

**Keywords:** Membrane biophysics, Membrane proteins

## Abstract

Sticholysins are α-pore-forming toxins produced by the sea-anemone *Stichodactyla helianthus*. These toxins exert their activity by forming pores on sphingomyelin-containing membranes. Recognition of sphingomyelin by sticholysins is required to start the process of pore formation. Sphingomyelin recognition is coupled with membrane binding and followed by membrane penetration and oligomerization. Many features of these processes are known. However, the extent of contact with each of the different kinds of lipids present in the membrane has received little attention. To delve into this question, we have used a phosphatidylcholine analogue labeled at one of its acyl chains with a doxyl moiety, a known quencher of tryptophan emission. Here we present evidence for the contact of sticholysins with phosphatidylcholine lipids in the sticholysin oligomer, and for how each sticholysin isotoxin is affected differently by the inclusion of cholesterol in the membrane. Furthermore, using phosphatidylcholine analogs that were labeled at different positions of their structure (acyl chains and headgroup) in combination with a variety of sticholysin mutants, we also investigated the depth of the tryptophan residues of sticholysins in the bilayer. Our results indicate that the position of the tryptophan residues relative to the membrane normal is deeper when cholesterol is absent from the membrane.

## Introduction

Sticholysins (Stns) are the preponderant toxins in the venomous cocktail of the Caribbean Sea anemone *Stichodactyla helianthus*^1–3^. These proteins belong to the actinoporin family of toxins and have a structure that consists of a β-sandwich flanked by two α-helices, as revealed by their three-dimensional structures^4–12^. One of these two helices, located at the N-terminal of the sequence, is ultimately responsible for pore formation and lining the lumen of the pore. Hence, actinoporins are classified as α-pore-forming toxins^12–18^.

Actinoporins, including Stns, exert their activity by forming pores on the membrane of the targeted cells, provided sphingomyelin (SM) is present and accessible for recognition^1,12,19,20^. The relatively straightforward selectivity displayed by actinoporins can be exploited to test their pore-forming ability on model membranes of the desired composition. This has been the main approach to delve into the behavior of these proteins, allowing us to resolve aspects of their behavior such as their membrane specificity and mechanism of membrane recognition^21–25^, membrane composition effects^26–32^, and the structure and stoichiometry of the final pore complex^6,8,10,33^. However, little is known about the detailed lipid environment that surrounds Stns once they are bound to the membrane. It has been shown that equinatoxin II (EqtII), an actinoporin from *Actinia equina*, induces reorganization of the plasma membrane^34^ and that StnII can reduce the contacts of cholestatrienol (CTL), a fluorescent analog of cholesterol (Chol), with PC lipids^35^. Still, the direct interaction of the pore complex with surrounding lipids remains poorly understood. The resolution of the fragaceatoxin C (FraC, from *A. fragacea*) pore by Tanaka et al. revealed the location of some lipids in the structure of the pore-lipid complex, as they were co-purified with the toxin ensemble^10^. At the time, those lipids were presumed to be SM, but the resolution of the structure solved did not allow for their unambiguous identification. Much more recently, new results obtained by molecular simulations have challenged that interpretation, indicating that SM and phosphatidylcholine (PC) would be equally likely to interact with these pore complexes when they are embedded in the membrane and have reached their thermodynamic equilibrium^36^.

**Figure 1:**
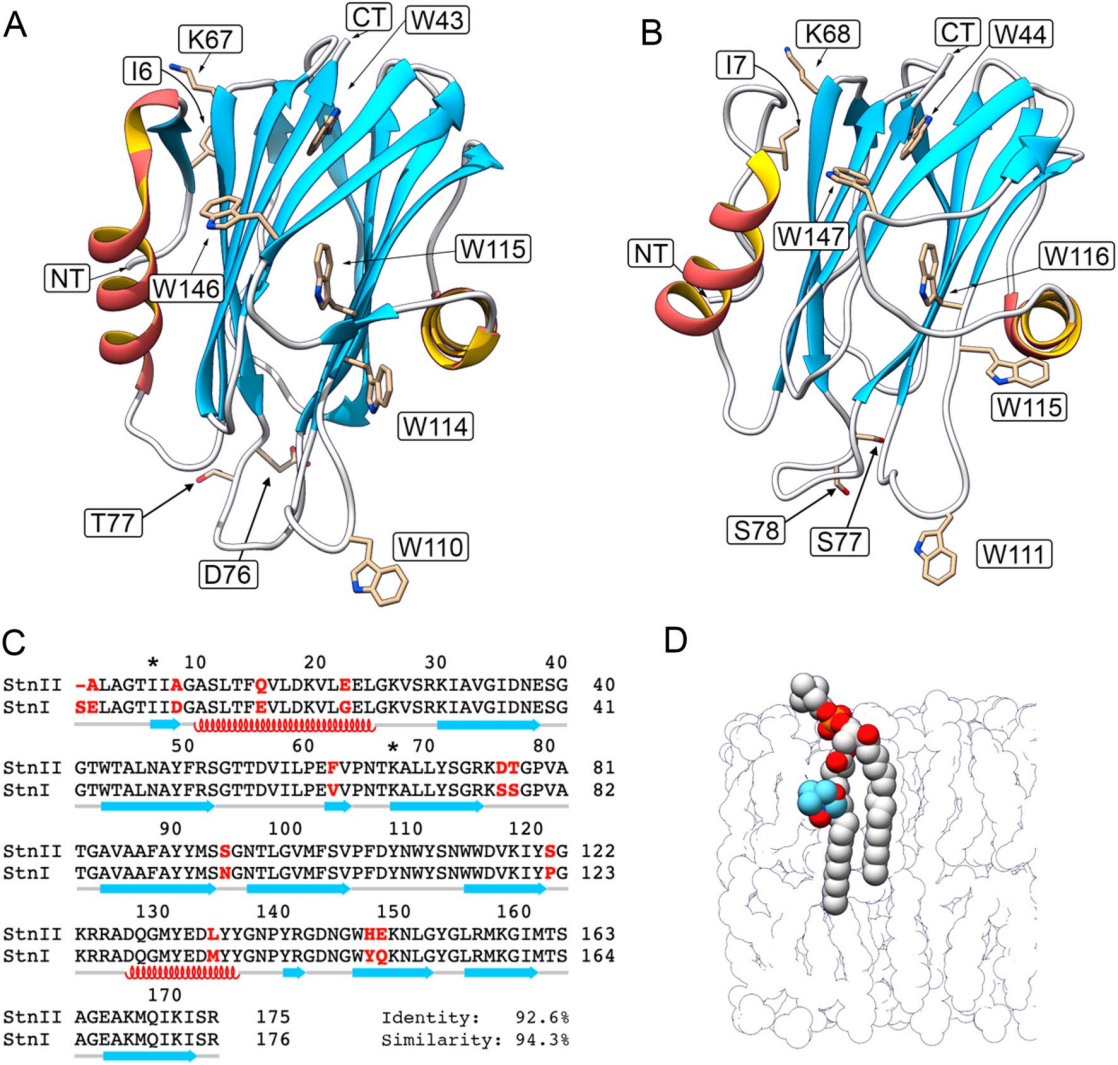
(**A**) The three-dimensional structure of StnII. PDB ID 1GWY. Indicated are all five Trp residues of the protein, plus I6 and K67, which have been mutated to Cys to form a disulfide bridge that prevents the deployment of the N-terminal α-helix. Of all five Trp residues, only W110 and W114 (W111 and W115 in StnI) are inserted in the membrane^35,37^. W43 and W115 are buried in the protein core, and W146 is known to participate in protein–protein interactions upon oligomerization^10,37,39^. The possible role of D76 and T77 is commented in the discussion. (**B**) The three-dimensional structure of StnI. PDB ID 2KS4. Indicated are the same features as in (**A**). (**C**) Sequence alignment of StnII and StnI. Shown are the secondary structure features of Stns. Colors are the same as in (**A**) and (**B**). Differences between StnI and StnII are highlighted in red. Asterisks indicate the positions mutated for this study. (**D**) Sketch of a bilayer in which a molecule of 7-SLPC has been highlighted. The doxyl moiety is shown in blue. Scale between (**A**) and (**B**) and (**D**) is approximately 1/2.

In the present work, we have evaluated the exposure of the Trp residues of StnI, StnII, and several of their mutants to PC lipids, using spin-labeled PC analogs. Sticholysins have five tryptophan (Trp, W) residues in their sequence. According to StnII numbering, these are W43, W110, W114, W115, and W146^35,37,38^ (Fig. 1). W43 and W115 are buried within the hydrophobic core of the structure, thus being shielded and essentially insensitive to environmental changes. W146 is exposed to the solvent in the monomeric soluble structure but, in the oligomer, it appears to be water-shielded due to monomer–monomer interactions within that ensemble^10,35,39^. Only W110 and W114 are solvent-exposed in the water-soluble structure and then embedded in the membrane once binding occurs^35,37^. Thus, it is essentially the emission of these two residues that is used as a reporter to monitor membrane binding from fluorescence emission. Two different membrane compositions have been used for the quenching experiments: DOPC:PSM 80:20, and DOPC:PSM:Chol 70:15:15. In these mixtures, a fraction of DOPC was replaced with different amounts of a spin-labeled PC, which should display a close enough behavior to that of DOPC, being in the fluid state at our experimental temperature (23 °C)^40,41^. Membranes with the first composition (80:20) are, according to Nyholm et al.^42^, in the *l*_*d*_ phase (Fig. S1). In principle, this would allow us to observe the accessibility of Trp residues to PC molecules when SM and PC are, essentially, uniformly mixed. Phase effects that could induce a preferential interaction with either lipid species should be negligible in this mixture. The second composition (70:15:15) was chosen because, as the standard lipid mixture used in the field, DOPC:SM:Chol 1:1:1, it allows for the coexistence of the *l*_*o*_ and *l*_*d*_ phases^42^, though, presumably, in a different proportion. The reason to use this composition instead of the standard is the much larger DOPC content (> 2x), which allows for a much broader range of quenching by DOPC substitution while, in principle, maintaining the relevant features of the toxin-membrane interaction. The comparison of these results with those obtained with 80:20 membranes could reveal if Trp residues are shielded from PCs by Chol and SMs, as hinted by our previous studies using CTL^35^.

Labeling at different positions of the acyl-chains of the PC analogs has allowed us to investigate the relative protein-lipid arrangement. Most often, depth-dependent quenching is used to resolve the position of a fluorophore within the membrane relative to the membrane normal^43–47^. However, this requires the structure of the membrane to be relatively unaltered, with the orientation of the lipid acyl-chains being approximately parallel to the membrane normal. If that arrangement is altered, probe depth cannot be determined, as label depth would not correspond to what has been previously measured.

It is unclear if pores formed by actinoporins, including sticholysins, are toroidal or not, since observations supporting both hypotheses have been made^6,10,48–50^. To further investigate this question, we used two double Cys mutants of StnI and StnII. These mutants are a rendition of the selectively active double Cys mutant of EqtII produced by professor Anderluh’s group^51–54^ that, when oxidized, is unable to deploy its N-terminal α-helix due to it being covalently bound to the β-sandwich. In absence of membrane penetration, the orientation of the labeled acyl-chains should still be about the same as in a toxin-free membrane. These results could then be compared with those obtained with the WT proteins, and even with those of Trp-mutants of StnII, to infer what changes on the membrane have occurred because of helix deployment.

## Materials and methods

### Materials

The lipids 1-palmitoyl-2-oleoyl-*sn*-glycero-3-phosphocholine (POPC), 1,2-dioleoyl-*sn*-glycero-3-phosphocholine (DOPC), *N*-palmitoyl-d-*erythro*-sphingosylphosphorylcholine (PSM), Chol, and all spin-labeled lipids (1-palmitoyl-2-stearoyl-(14-doxyl)-sn-glycero-3-phosphocholine (14-SLPC), 1-palmitoyl-2-stearoyl-(12-doxyl)-sn-glycero-3-phosphocholine (12-SLPC), 1-palmitoyl-2-stearoyl-(10-doxyl)-sn-glycero-3-phosphocholine (10-SLPC), 1-palmitoyl-2-stearoyl-(7-doxyl)-sn-glycero-3-phosphocholine (7-SLPC), 1-palmitoyl-2-stearoyl-(5-doxyl)-sn-glycero-3-phosphocholine (5-SLPC), and 1,2-dioleoyl-*sn*-glycero-3-phospho(tempo)choline (Tempo-PC)) were acquired from Avanti Polar Lipids (Alabaster, AL, USA). Disuccinimpidyl suberate (DSS) was from Thermo-Fisher Scientific (Waltham, MA, USA). All sticholysin variants were produced in *E. coli*, strain RB791, and purified to homogeneity as described in^37,55^.

### Methods

#### Protein production and characterization

All sticholysin mutants were produced using site-directed mutagenesis. The design of the double Cys mutants was a rendition of an EqtII mutant by professor Anderluh’s group^51–54^, as stated above.

The new mutants, StnI-I7C/K68C and StnII-I6C/K67C were characterized functionally and structurally using circular dichroism, intrinsic fluorescence emission, and hemolytic assays, as previously described^56^. The hemolytic activity of the mutants was negligible when oxidized (hereon indicated as StnI-I7C/K68C^ox^, for example). However, if the disulfides were reduced before exposing the toxin to the erythrocytes, the hemolytic activity was almost completely recovered to the values of the WT variant (Fig. S2).

#### Vesicle preparation

Dry lipid films were prepared from the chosen organic lipid solutions combined in the desired molar proportion, followed by evaporation of the organic solvent under nitrogen flow at 40 °C. These films were then hydrated in buffer (10 mM Tris, 140 mM NaCl, pH 7.4) at 65 °C in a water bath for at least 30 min and suspended by gentle vortex. This method yields symmetric bilayers arranged in multilamellar vesicles, with all lipid species randomly distributed between the leaflets. Suspended lipid vesicles were then extruded at hydration temperature through polycarbonate filters to obtain large unilamellar vesicles (LUVs) of 200 nm average size. According to the manufacturer (Avanti Polar Lipids^57^), this system is expected to yield samples with low polydispersity, if filters with pore size ≤ 200 nm are used. DLS measurements have confirmed this previously^58^ and for the current samples (data not shown). Vesicle symmetry is expected to be maintained in the time between sample preparation and experiment. Lipid content of LUV stocks was measured after the experiments by phosphorous quantitation according to the method of Rouser^59^.

#### Crosslinking experiments

Crosslinking experiments were performed to study the oligomerization state of the inactive Stn mutants StnI-I6C/K67C^ox^ and StnII-I7C/K68C^ox^. The crosslinking agent was DSS, reactive with amino groups and with a spacer arm length of only 11.4 Å. Briefly, LUVs of the chosen composition were incubated with toxin at 23 °C for 30 min to ensure complete binding. Small aliquots of freshly prepared crosslinker dissolved in DMSO were added to a final DSS/P ratio of 40. [DMSO]_sample_ was < 2.5% (v/v) to minimize possible effects on proteins or membranes. A lipid/protein (L/P) ratio of ~ 180 (1.6 mM and 9 µM, respectively) was used to hinder the potential crosslinking of non-interacting protomers. One can estimate the mean distance between monomers (or oligomers) based on their surface density^33,60^, as ${r}_{av}=\frac{\sqrt{\sigma }}{2}$, where *r*_*av*_ is the average distance between the particles, and *σ* is their surface density. This can be estimated from the L/P ratio, and the average lipid cross-sectional area. We used average cross-sectional areas values of 45 – 60 Å^2^, depending on the membrane composition (see^61–64^ for lipid coss-sectional areas). At this L/P ratio, the mean distance between free monomers on the membrane surface should be at least ~ 25 Å if the radius of the protein is also considered, being larger in less condensed membranes, with less or no Chol. Mean distance would increase to > 100 Å for octamers. Based on time scans performed using StnII WT with DOPC:PSM:Chol 1:1:1 as control (Fig. S3a), the reaction time with DSS was 10 min. The reaction was stopped using a 2 M solution of glycine, added to a final Gly/DSS ratio of 126, followed by 10 min incubation. Electrophoresis loading buffer with 0.5% (v/v) β-mercaptoethanol was added, and the samples were boiled for 20 min. Cross-linked products were analyzed using standard PAGE-SDS procedures^65^. Control experiments were also performed in absence of membranes to test if DSS produced intra-molecular adducts that affected the electrophoretic mobility of the toxins (Fig. S3b).

#### Time-resolved fluorescence measurements

Time-resolved fluorescence measurements were performed using a FluoTime100 spectrofluorimeter, equipped with a PicoHarp300E time-correlated single photon-counting module (PicoQuant GmbH, Berlin, Germany). A 297 ± 15 nm pulsed diode laser was used for excitation. Emission was collected through a long-pass filter (> 345 nm) to avoid stray light. When required, neutral density filters were used to attenuate excitation intensity. The instrument response function (IRF) was acquired using light scattered by a LUDOX solution in absence of colored filters up to ~ 10,000 counts in the peak channel. Sample decays were recorded up to ~ 12,000 counts in the peak channel. Inner filter effects were avoided by using fluorophore concentrations such that the optical density at the excitation wavelength was OD_*l*/2_ < 0.05. Constant stirring was maintained during all experiments. A Peltier element was used to ensure steady temperature. Details on the analysis are provided in the supporting information (S.I.) file.

#### Steady-state fluorescence spectroscopy

Steady-state fluorescence measurements were performed on a PTI Quanta-Master spectrofluorimeter (Photon Technology International, Lawrenceville, NJ, USA). Sample excitation was 295 nm to specifically excite Trp residues. Emission was recorded between 305 and 450 nm, at 1 nm intervals. Polarizers were used to minimize light scattering in the signal and to avoid distortions in the acquired spectra. The configuration was Em_pol_ = 0°, Ex_pol_ = 90°, as recommended these ends in^66^. Again, inner filter effects were avoided by using fluorophore concentrations such that OD_*l*/2_^λex^ < 0.05. Experiments were performed in PBS (10 mM phosphate, 140 mM NaCl, pH 7.4). Constant stirring was used. The temperature was controlled by a Peltier element. When the temperature effect was evaluated, the gradient was 5 °C/min. The fluorescent signal was corrected for inner and outer filter effects produced by vesicle-induced light scattering, as indicated in^66^, using the same amounts of toxin and lipids as in the original experiments, but with LUVs composed solely of POPC, to which no toxin binding could be detected. Details on the analysis of the shape of the emission spectra of Trp are provided in the S.I. file.

#### Toxin titration with quencher-free vesicles

To properly evaluate Trp quenching, complete membrane binding by the toxins was required. All toxin variants (100 nM) were titrated with quencher-free vesicles of the desired compositions to saturation. The results were used to estimate the fraction of bound protein as a function of lipid and protein content in the sample. Analysis was performed according to

1 $$\theta \left(\left[{L}_{T}\right],\left[{P}_{T}\right]\right)= 1-\frac{1}{2}\left[1-\frac{\left[{L}_{T}\right]}{n\left[{P}_{T}\right]}-\frac{1}{{K}_{a}\left[{P}_{T}\right]}+ \sqrt{{\left(\frac{\left[{L}_{T}\right]}{n\left[{P}_{T}\right]}+\frac{1}{{K}_{a}\left[{P}_{T}\right]}-1\right)}^{2}+\frac{4}{{K}_{a}\left[{P}_{T}\right]} }\right],$$

as described in^67,68^. In this equation, θ is the fraction of bound protein, which is a function of the total lipid and protein concentrations, [*L*_*T*_] and [*P*_*T*_] respectively. The values of [*L*_*T*_] and [*P*_*T*_] were corrected for dilution effects caused by LUV addition to the sample. *K*_*a*_ is the association constant, and *n* is the stoichiometry of the interaction, understood as one monomer of protein binding to LUVs every *n* lipid molecules (without any implication on the possible direct interactions).

In all cases, goodness of fit was evaluated, at least, according to the residuals trace, the root mean squared deviation (RMSD), and the value of χ^2^_red_.

#### Analysis of tryptophan exposure to lipids

The exposure of a membrane-bound fluorophore to surrounding lipids can be studied using fluorescence quenching^40,69–73^. Using increasing amounts of quencher-labeled lipids in the membrane, the number of boundary lipids that a membrane fluorophore is in contact with can be estimated from

2 $$F\left(\left[\mathrm{Q}\right]\right)= \left({F}_{0}-{F}_{min}\right){\left(1-\left[\mathrm{Q}\right]\right)}^{{L}_{n}}+{F}_{min},$$

where *F*([Q]) is the fluorescence signal measured at a given mol% of quencher in the membrane, *F*_0_ is the emission of the sample in absence of quencher, *F*_*min*_ is the intensity observed when the protein is in pure quencher lipid, and *L*_*n*_ is the number of lipids that can establish direct contact with the studied fluorophore. *F*([Q]) was normalized as *F*/*F*_0_. Detail of the analysis is provided in the S.I. file.

#### Depth-dependent fluorescence quenching of tryptophan

The position of a membrane-embedded fluorophore along the membrane normal can be estimated using quencher-labeled lipids in which the quencher group is located at varying positions along the acyl chains of the lipids, or at the headgroup^43–47,74–77^. The fluorescence intensity of the sample, or the lifetime, can be measured in the absence and presence of each of the quenchers. The depth-dependent quenching profile, *QP*(*h*), defined as *QP*(*h*) = *F*_0_/*F*–1 for intensity measurements^77^, can be obtained from these data. The *QP*(*h*) can be approximated, according to the distribution analysis, with two symmetrical Gaussian functions, as

3 $$QP\left(h\right)=\frac{S}{\sigma \sqrt{2\pi }}\mathrm{exp}\left[-\frac{\left(h-{h}_{m}\right)}{2{\sigma }^{2}}\right]+ \frac{S}{\sigma \sqrt{2\pi }}\mathrm{exp}\left[-\frac{\left(h+{h}_{m}\right)}{2{\sigma }^{2}}\right],$$

where *h*_*m*_ is the center of the profile measured from the bilayer center (*h* = 0), indicating the average depth of the fluorophore in the membrane; *σ* is the distribution width, and corresponds to the fluctuations in *h*; and *S* is the area, related to fluorophore accessibility. The bilayer center is the symmetry axis of the mirrored Gaussian functions, which are required to account for trans leaflet quenching on deep fluorophores^43^. Values of membrane quencher depths from the bilayer center were 18.2 (TEMPO-PC), 12.1 (5-SLPC), 11.5 (7-SLPC), 10.1 (10-SLPC), 6.4 (12-SLPC), and 2.9 Å (14-SLPC), from^78^, where these were obtained in pure POPC bilayers, being the best available approximation.

## Results

### Toxin titration with quencher-free vesicles

All toxins were titrated with quencher-free vesicles of each of the selected compositions. This allowed us to estimate the stoichiometry of each toxin-membrane interaction (Table 1 and Fig. S4). In all cases, based on fluorescence emission of each toxin variant, saturation is reached at L/P molar ratio < 80. Thus, for subsequent experiments, a L/P molar ratio of 120 was judged as enough to ensure complete binding in all cases. At the L/P ratio, the average distance between free monomers would be ~ 25 Å, increasing to ~ 100 Å if octameric pores are formed (not considering the surface corresponding to the lumen of the pores).

**Table 1:** Affinity (*K*_a_), stoichiometry (*n*) of the interaction, and increase in emission (F_b_/F_sol_) of WT sticholysins and several of its mutants upon interaction with membranes of different compositions. Values of *n* and *K*_a_ are obtained from fitting using non-linear least squares. Note that the stoichiometry, *n*, is understood as a protein monomer binding the membrane every *n* lipids, not necessarily interacting directly with all of them. Values are average ± SEM (n = 2–3).

	DOPC:PSM:Chol 70:15:15			DOPC:PSM 80:20		
	*n*	*K*_a_ (× 10^8^ M^−1^)	F_b_/F_sol_	*n*	*K*_a_ (× 10^8^ M^−1^)	F_b_/F_sol_
StnI	55.3 ± 0.6	4.1 ± 0.0	1.75 ± 0.01	63.2 ± 3.5	1.1 ± 0.0	1.45 ± 0.01
StnI-I7C/K68C^ox^	67.7 ± 0.3	4.2 ± 0.0	1.94 ± 0.02	54.2 ± 3.3	7.5 ± 0.0	1.56 ± 0.00
StnII	58.1 ± 0.4	6.7 ± 0.0	1.72 ± 0.01	60.8 ± 0.1	0.4 ± 0.0	1.25 ± 0.01
StnII-I6C/K67C^ox^	61.5 ± 1.6	9.1 ± 0.1	1.84 ± 0.00	42.1 ± 0.9	2.1 ± 0.0	1.50 ± 0.00
StnII-W43/110F	67.9 ± 2.2	1.2 ± 0.0	1.73 ± 0.00	60.0 ± 5.9	0.5 ± 0.0	1.28 ± 0.01
StnII-W43/114F	79.1 ± 1.6	6.1 ± 0.0	1.74 ± 0.01	–	–	–

It is interesting to compare the stoichiometry and affinity of the non-competent mutants, StnI-I6C/K67C^ox^ and StnII-I7C/K68C^ox^, with that of the WT variants. In presence of Chol, when the *l*_d_ and *l*_o_ phases are present, the parameters obtained are very similar for all variants of each toxin isoform, with the stoichiometry of the mutants being slightly higher. Only StnII-I6C/K67C^ox^ displays a higher affinity than its WT counterpart. Unexpectedly, when Chol is absent and the membrane is in the *l*_d_ phase, the affinity of these mutants is much larger (~ 6x) than that of the corresponding WT variant, despite their inability to deploy the N-terminal α-helix.

Regarding the Trp residues, W114 seems to play a much more relevant role than W110 in maintaining binding affinity, despite W110 being more solvent exposed in the soluble structure. StnII-W43/110F behaves much like StnII-WT, just with a somewhat reduced membrane affinity toward Chol-containing membranes. However, the substitution of W114 in mutant StnII-W43/114F results in a toxin that can bind membranes with Chol but whose Trp emission is not increased when titrated with Chol-lacking membranes.

In all cases, the increase in fluorescence emission, calculated as F_bound_/F_solution_ for each variant (Table 1), is much larger when Chol is present in the membrane.

### Oligomerization state on the membrane of the inactive mutants

**Figure 2:**
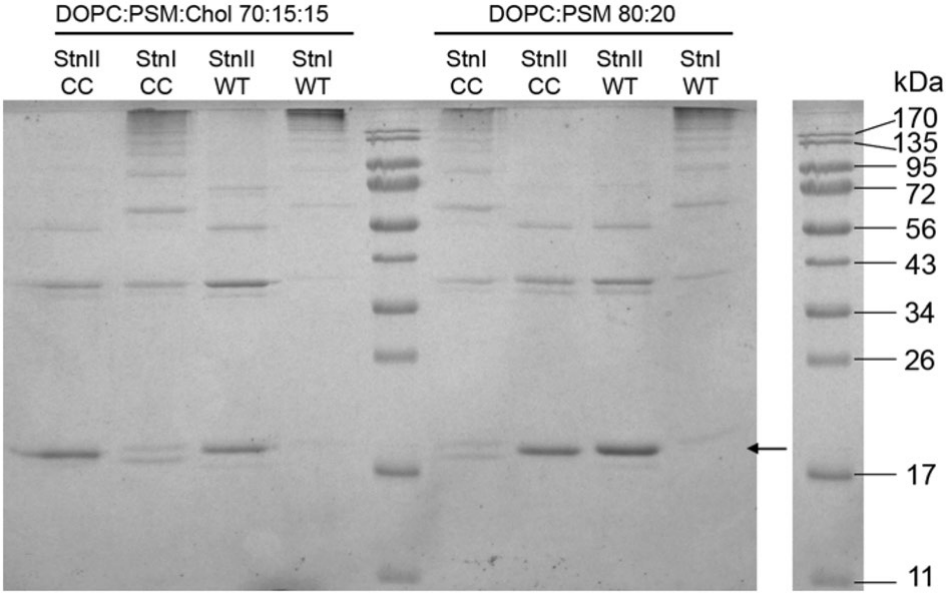
Results of the crosslinking experiments. The indicated Stn variant (CC indicates oxidized double-Cys mutant) was first incubated with LUVs of the indicated composition for 30 min, and then with 10 min with DSS. The expected position of the monomers (~ 19 kDa) in indicated with a small arrow. M_w_ of the proteins in the ladder are shown on the right.

To investigate the oligomerization state of StnI-I7C/K68C^ox^ and StnII-I6C/K67C^ox^ when bound to the membrane, crosslinking experiments were performed. Results show that both mutants behave essentially as the corresponding WT protein (Fig. 2**)**. This suggests that the conformation of the N-terminal α-helix is not relevant regarding adduct formation during the crosslinking reaction.

The differences between isoforms probably reflect their distinct reactivity with DSS. The split bands observed at the expected position of the monomers (Fig. 2) together with the occurrence of the same bands in the controls (Fig. S3b) indicate that StnI is more prone to form intramolecular adducts and, overall, more reactive. Hence, exposure of oligomers to DSS would result in faster crosslinking and, consequently, higher abundance of low mobility adducts, as revealed by the different profiles observed for StnI and StnII and their corresponding mutants shown in (Fig. 2).

### Quenching of tryptophan emission by spin-labeled lipids: Effects on membrane-binding and Trp emission intensity, shape, and lifetime: effect of temperature

The emission at 330 nm under 295 nm excitation was recorded to test the effect of spin-labeled lipids on membrane binding. In all cases, a time-dependent increase in the emission as a response to lipid addition to the sample was observed (Fig. S5). The kinetic parameters of the interactions of each toxin variant with vesicles of each composition (Fig. S6) show that all Stn variants were faster in Chol-containing membranes. In these cases, the dominant component was the fastest one (*α*_1_ >  ~ 0.8; *τ*_1_ < 5 s, typically). A second component (with *τ*_2_ ~ 50–70 s) was also detected in all cases. Increasing the mol% of 7-SLPC in membranes with Chol did not significantly perturb the kinetics. Only those of StnII-W43/114F appear to be affected by the quencher (Fig. S6).

The binding process to membranes without Chol was slowed down by increasing 7-SLPC. The fastest component was observed to become less dominant. Interestingly, this did not occur for the inactive mutants, for which only the secondary process seemed to be affected.

To ensure that the same degree of binding was achieved regardless of the presence of 7-SLPC, the shapes of the emission spectra (analyzed using eq. S2.) were compared. These spectra were recorded after the time-dependent signal had plateaued. Only a small quencher-dependent broadening (< 2 nm) and red-shift (0–2 nm) of the spectra was observed for the WT, the inactive mutants, and StnII-W43/110F in Chol-containing membranes (Fig. S7). In the other instances, the emission of the Trp mutants was broadened and red-shifted (~ 10 nm and ~ 6 nm, respectively). The combination of spectral and kinetic data suggests that, while increased 7-SLPC content in the membrane can affect membrane binding, it only slows down the process, without preventing it. The spectral differences observed are likely a consequence of the lost contribution of the quenched Trp residues to the total spectra of the toxin, which maintain the emission of the shielded Trp.

Quenching of Trp emission by spin-labels has been shown to be essentially collisional rather than a static process^46,76,79^. However, given that the diffusion coefficients of lipids in membranes are 10^–7^–10^–8^ cm^2^ s^–1^^80^, molecules with an excited state lifetime < 50 ns would experience less than one collision during the excited state unless already in contact with the quencher^40^. In fact, integral membrane proteins appear to diffuse together with annular lipids^81^, reducing the effective diffusion coefficient of those lipids. Hence, the observed quenching would appear as static quenching, even if the intrinsic nature of the process is collisional. This is the basis of the model with which the number of lipids that surround a fluorophore can be estimated through fluorescence quenching^40,70,71^.

**Figure 3:**
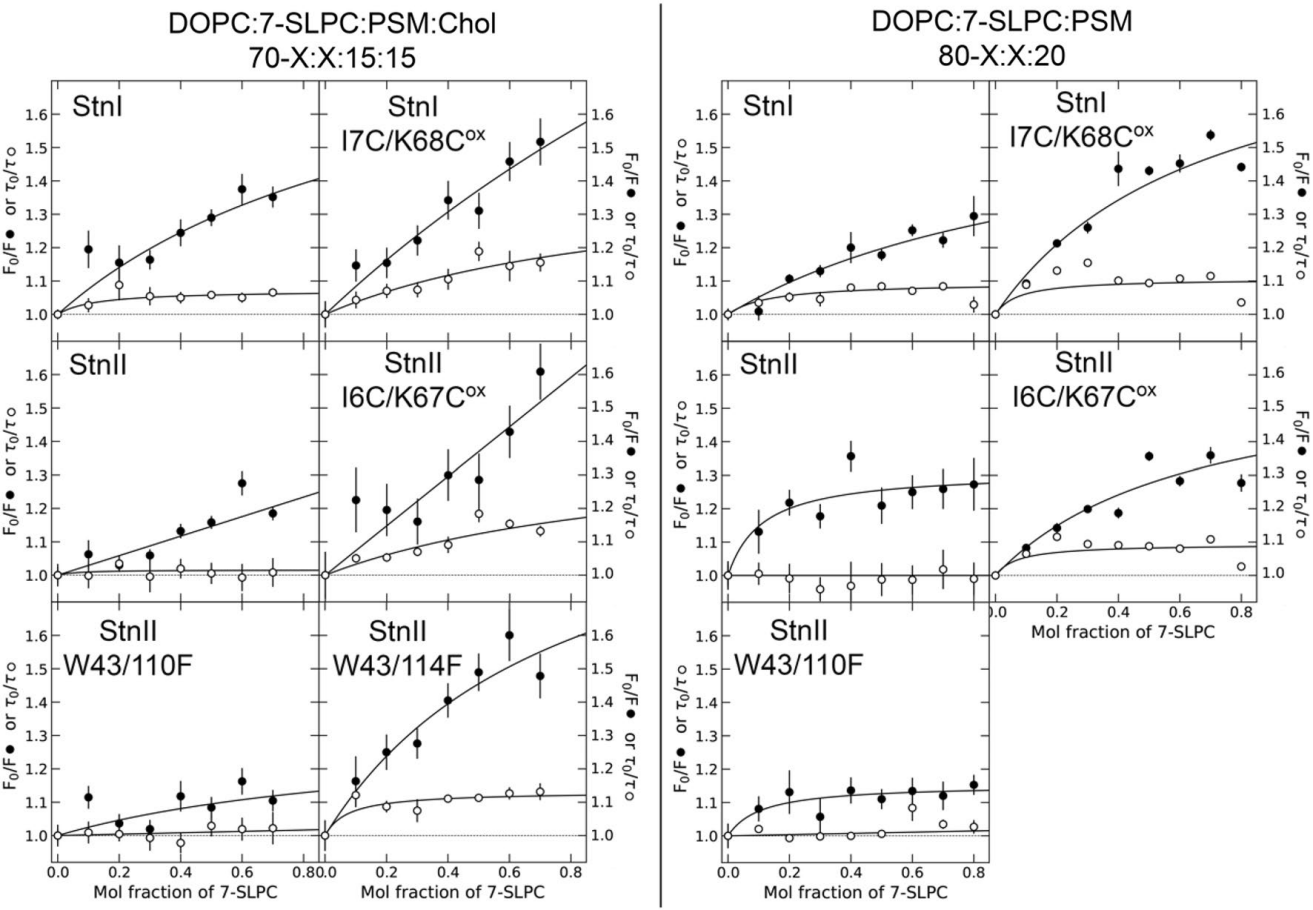
Fluorescence quenching in membranes. Quenching of fluorescence emission of Stn variants by increasing amounts of 7-SLPC in membranes composed of DOPC:7-SLPC:PSM:Chol (left side panels) or DOPC:7-SLPC:PSM (right side panels) in molar relations of 70-X:X:15:15, or 80-X:X:20, respectively, where X is the molar fraction of 7-SLPC. Since StnII-W43/114F cannot bind the Chol-lacking membranes used, the analysis could not be done with this membrane composition. Closed symbols: F_0_/F (steady state). Open symbols: τ_0_/τ (lifetime data). Data are average ± SEM of n = 2–3. Lines are guides to the eye. In all cases, RMSD was < 0.04, and χ^2^_red_ < 1.38.

To test quenching of Stn’s Trp by spin-labels, the emission of the sample was measured using steady-state and time-resolved spectroscopy. Three lifetime components of about 8, 3, and 1.5 ns were required to obtain satisfactory fits to the fluorescence intensity decays. A plot of τ_0_/τ (intensity-weighted average lifetimes) obtained without (τ_0_) and with increasing amount of quencher (τ), revealed, in most cases, a dynamic component of quenching (Fig. 3). However, the F_0_/F values obtained using steady-state data are much larger, indicating that quenching occurs, mainly, by a process equivalent to static quenching. In nearly all cases, results displayed a downward curvature, away from the *y* axis, with increasing mol% of 7-SLPC. This deviation from the typical Stern-Vollmer behavior is expected when only part of the total fluorophore population is accessible to the quencher, as in Stns (see Fig. 1)^69,82,83^.

Lifetime data indicates that collisional quenching is minimal or does not occur in the situations studied. However, given that all Stn variants used have three or more Trp residues, and that even single-Trp proteins often display multiple exponential decays^69,84,85^, it was possible that the shielded Trp residues could mask the reduced lifetime of the quencher-exposed Trp residues. Hence, given that collisional quenching increases with temperature, the steady-state emission was also recorded for the samples containing 30 and 70 mol% of 7-SLPC while increasing the temperature of the sample (Figs. S8 and S9). In most cases for Chol-containing membranes, quenching increased with temperature, though not steadily. This suggests that, while collisional quenching may occur, it depends on membrane organization around the protein complexes, with the main mechanism being static. In absence of Chol, quenching does not vary, and, in some cases, it even diminishes with increasing temperature. Rearrangements in the vicinity of the toxin oligomers may diminish Trp-quencher contacts.

### Tryptophan exposure to PC lipids

Given that most of the quenching observed for all Stn variants used could be interpreted as static quenching, we proceeded to analyze the steady-state data presented in Fig. 3 according to Eq. 2. These analyses (Figs. S10 and S11) allowed us to estimate the fraction of the fluorophore population that is inaccessible to quencher and, most importantly, the number of PC lipids in contact with accessible Trp residues (Table 2).

**Table 2:** PC-lipid contacts. Number of PC lipids (*L*_*n*_) in contact with the membrane inserted Trp residues of the indicated Stn variants in membranes with and without Chol. The value of the non-quenchable fluorescence (*F*_*min*_) is also indicated. Values are result ± SD, obtained by fitting procedures.

	DOPC:PSM:Chol 70:15:15		DOPC:PSM 80:20	
	*L*_*n*_	*F*_*min*_	*L*_*n*_	*F*_*min*_
StnI	2.49 ± 0.86	0.72 ± 0.03	2.38 ± 0.67	0.79 ± 0.02
StnI-I7C/K68C^ox^	2.16 ± 0.87	0.64 ± 0.06	3.26 ± 0.25	0.67 ± 0.01
StnII	1.15 ± 0.69	0.76 ± 0.09	7.15 ± 3.88	0.79 ± 0.02
StnII-I6C/K67C^ox^	1.22 ± 0.91	0.53 ± 0.19	3.06 ± 0.40	0.75 ± 0.01
StnII-W43/110F	2.40 ± 2.42	0.89 ± 0.05	9.34 ± 9.72	0.89 ± 0.02
StnII-W43/114F	3.20 ± 1.03	0.64 ± 0.04	–	–

Results indicate that, for most Stn variants, the inaccessible fluorophore population would be responsible for 60–75% of the Trp emission. StnII-W43/110F is an exception, showing that W114 is mostly protected from 7-SLPC.

Trp accessibility results are most interesting. StnI appears to be exposed to 2.5 PC lipids in both membrane systems studied. However, the presence of Chol greatly affects the accessibility of StnII’s Trp. When Chol is absent, StnII has contact with > 4 PCs. However, in Chol-containing membranes, only ~ 1 PC molecule can reach the Trp residues of StnII. The inactive mutants show the same behavior as the corresponding WT protein. The values obtained for StnII-W43/110F are not very reliable, possibly due to the low resolution allowed by the small effect of 7-SLPC on its emission. However, the results suggest that W114 is much more accessible to 7-SLPC in Chol-lacking membranes, given that the corresponding curve becomes saturated at just 20 mol% of 7-SLPC. Since StnII-W43/114F cannot bind the Chol-lacking membranes used in this work, the analysis could only be done in membranes with Chol. It appears that W110 can be in contact with ~ 3 PC molecules. Given that the WT protein’s Trp would only be in contact with ~ 1 quencher, this suggests that W114 establishes lipid interactions with Chol and/or PSM that reduce PC abundancy in the surroundings of the pore structure. This observation would support the inaccessibility of W114.

### Position of the Trp residues of Stns relative to the membrane normal

Quenching experiments using spin-labeled lipids that differ from each other in the position of the quencher group were performed to estimate the membrane depth of the Trp residues of Stns. For this, 30 mol% quencher lipid was included in the corresponding membrane system.

The lipid arrangement in the pores formed by actinoporins is unclear. Toroidal and non-toroidal architectures have been proposed^10,32,86,87^. Hence, since the depth of the quenchers has been measured for undistorted membranes^46,47,77^, the success of this approach depended on the existence of a membrane distortion induced by a toroidal architecture and the extension of this effect.

**Figure 4:**
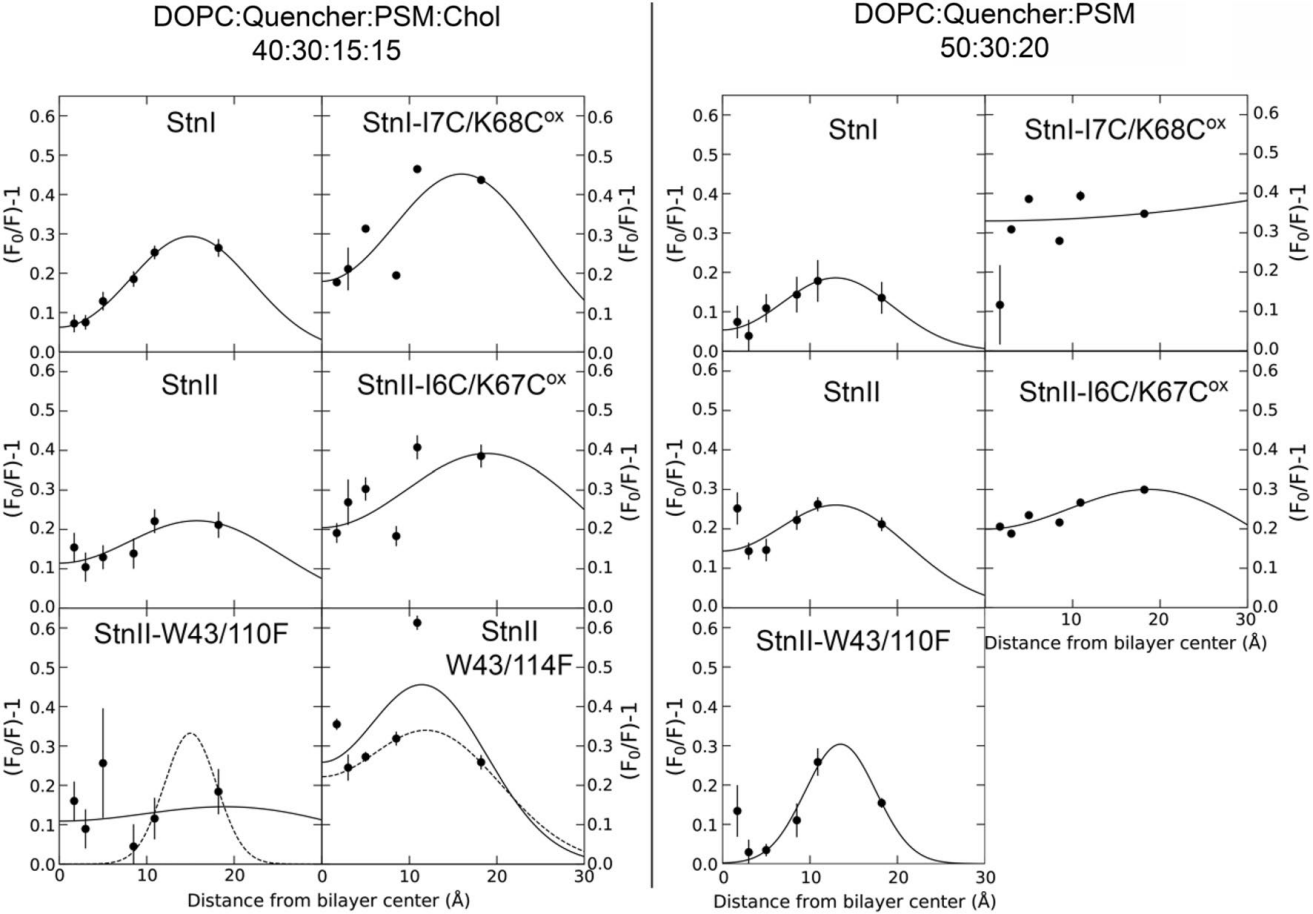
Depth-dependent quenching profiles of Stns in membranes. The obtained profiles represent the most probable depths at which the membrane embedded Trp residues of the indicated toxin variant are found in membranes composed of DOPC:Quencher:PSM:Chol (left side panels) or DOPC:Quencher:PSM (right side panels) in molar relations of 40:30:15:15, or 50:30:20, respectively Solid lines are best fits to Eq. 6. Dashed lines are fits of an alternative analysis in which some of the data has been discarded. This was done on grounds of (1) data deviations from the trend and/or (2) consistency with results obtained for other variants or for the same variant in the other membrane system. Quenching values are average ± SEM of n = 2–3.

Results shown in Fig. 4 suggest that the lipid acyl-chains still are more or less parallel to the membrane normal, at least in the vicinity of the Trp residues of the competent proteins. It appears that the average position of the Trp residues of the WT proteins is slightly deeper (~ 2 Å) when Chol is absent from the membrane, according to fitting results (Table 3). Though not as clearly, this also occurs to StnII-W43/110F. Results from the Trp mutants indicate, as expected, that W110 is placed deeper in the membrane than W114. Not only that, but W110 also seems to be much less protected by either the protein structure or by other lipids.

**Table 3:** Fitting results of the depth-distribution analysis. Values were obtained from fitting Eq. 6 to the quenching results shown in Fig. 4. Parameters are average depth (*h*), width of the distribution (σ), and area (*S*), related to fluorophore accessibility. Indicated data for StnII-W43/110F in presence of Chol were obtained using data from Chol-lacking membranes as guidance since the initial fit was unsuccessful. Data shown for StnII-W43/114F are from the dashed line fit in Fig. 4. However, results are very similar for the fit shown with solid lines.

	DOPC:Quencher:PSM:Chol 40:30:15:15			DOPC:Quencher:PSM 50:30:20		
	*h* (Å)	σ (Å)	*S* (Å)	*h* (Å)	σ (Å)	*S* (Å)
StnI	15.0 ± 1.0	7.1 ± 0.8	5.2 ± 0.2	12.9 ± 2.2	6.6 ± 2.2	3.1 ± 0.8
StnI-I7C/K68C^ox^	16.0 ± 0.3	8.9 ± 0.2	10.1 ± 0.2	–	–	–
StnII	15.6 ± 4.7	9.6 ± 3.6	5.3 ± 1.9	13.1 ± 1.1	8.2 ± 1.1	5.3 ± 0.5
StnII-I6C/K67C^ox^	19.0 ± 5.7	11.6 ± 3.6	11.4 ± 4.4	19.2 ± 2.2	13.0 ± 1.4	9.7 ± 1.3
StnII-W43/110F	15.5 ± 3.3	3.8 ± 4.7	2.3 ± 0.8	13.5 ± 0.4	4.0 ± 0.6	3.1 ± 0.4
StnII-W43/114F	12.2 ± 0.7	8.2 ± 1.2	6.9 ± 0.5	–	–	–

Data from the inactive mutants is not so clear, but still seems to support that acyl-chain orientation is not affected near the Trp side chains. In agreement with results from previous sections, the Trp residues of the double-Cys mutants seem to be more exposed to the quenchers. Again, this seems reasonable given that, for these proteins, the volume that corresponds to the pore-lumen in the rest of the toxins is not devoid of lipids nor occupied by the N-terminal α-helices of these proteins.

## Discussion

In this study, the lipid environment of the membrane-embedded Trp residues of Stns has been examined in membranes with and without Chol. We began by studying the interaction of six different Stn variants, two WT, and four mutants, with membranes of two different compositions, DOPC:PSM 80:20 and DOPC:PSM:Chol 70:15:15.

As observed for other lipid compositions^37^, the substitution of W114 by Phe results in a mutant (StnII-W43/114F) that is essentially unable to bind to Chol-lacking membranes. Given the different phase state of the two chosen compositions (see Fig. S1), it could be hypothesized that W114 plays a role in sensing the phase state of the membrane. The fact that the absence of W114 prevents membrane binding when Chol is absent too suggests that either Chol or W114 are required for the recognition of the SM-headgroup. In fact, W116 of EqtII has been predicted to participate in a cation-π interaction with the SM-headgroup^25^. This interaction should be present whenever SM is present in the membrane. The relevance of this interaction is highlighted by StnII-W43/110F, and not StnII-W43/114F, being capable of binding DOPC:PSM 80:20 membranes.

The larger increment in the fluorescence emission when the membranes used contain Chol, and this being even larger for the inactive mutants, is also revealing. This result is probably a consequence of the acyl-chain order near the Trp residues. Lipids in the *l*_d_ phase allow more water molecules across the membrane^88–90^, which can result in fluorescence quenching by water despite the toxins being membrane-bound. Given that the Chol-containing membranes used in this study are in a mixture of *l*_d_ and *l*_o_ phases, this result also suggests that pores in these membranes find themselves in an environment that is more like the *l*_o_ phase than the *l*_d_ phase in which the Chol-lacking membranes are. Following the water-quenching argument, the further increased emission of the oxidized double Cys mutants could result from the absence of a pore lumen, regardless of the oligomerization state of these toxin variants. The Trp residues of these mutants would be less accessible to water since the membrane integrity is less affected by their binding than by that of a fully functional Stn that penetrates the membrane with its N-terminal α-helix.

Regarding the observed binding kinetics reported by Trp emission, the fastest resolved component could be attributed to the water-membrane transition, whereas the slower one could be related to oligomerization and the rearrangements leading to the final pore structure. The main effect of increasing 7-SLPC in membranes without Chol on the binding kinetics was reducing the amplitude of the fast component. This could be due to a diminished initial raise in fluorescence emission, which occurs in absence of quencher by the solvent-to-membrane transition of the toxins. This effect would be smaller in Chol-containing membranes, as the exposure to PC lipids is also diminished in that case.

Though minimal in most cases, quencher-induced changes in the shape of the emission spectra were significant for StnII-W43/114F and StnII-W43/110F. Since a plot of FWHM vs λ_max_ of these data (Fig. S7) follows the same trend as the data obtained by titration with quencher-free vesicles (data not shown), we observe that the spectra are essentially the same if W110 and/or W114 are quenched, regardless of whether it is by water or by 7-SLPC. Not only that, but also that W110 and W114 are the only residues affected by the presence of 7-SLPC.

Despite Trp quenching by spin-labels being of dynamic nature^46,76,79^, for Stns, this shows itself as static quenching. Despite the intrinsic nature of the mechanism being dynamic, observed quenching can still appear as static if the quencher participates in a molecular complex in which the residence time of the components is long enough so that replacement of these molecules is unlikely to happen during the excited state of the fluorophores involved. The fact that in some cases quenching diminishes with increasing temperature suggests looser complexes that can dissociate with the effect of temperature. In fact, the traces that show a reduction in quenching are precisely those of StnI-I7C/K68C^ox^ and StnII-I6C/K67C^ox^, precisely the mutants whose interaction with lipids might not be as tight given their inability for membrane penetration. This would allow for an easier rearrangement, with a possible preference towards SM, given the higher T_m_ of this lipid.

The measured exposure of the Trp residues of Stns is interesting. Our results in absence of Chol would support recent molecular dynamics simulations showing that the lipid environment of FraC octamers would not display a preference for SM over PC^36^. The situation when Chol is present might be different. In principle, if Stns did not display any selectivity, results should be about the same regardless of membrane composition. However, if specific lipid-protein interactions that prevent potential quencher-Trp contacts are favored, results should reflect the presence of the preferred lipid in the membrane. In this regard, the behavior of StnI and StnII seems to be strikingly different. While StnI-quencher contacts are apparently the same in both membrane systems used, a very different situation is found for StnII, which is much more accessible to PC lipids when Chol is absent in the membrane. This is most likely an outcome of Chol standing in the way of the quencher, perhaps in an interaction also involving SM. This would agree with previous observations^35^, supporting past results indicating that the activity of StnII is much more favored by Chol than StnI’s^24,91,92^. Interestingly, this behavior, though helpful, in principle, for pore formation, appears to be independent of helix deployment since the inactive mutants appear to establish the same interactions as the WT proteins. A combination of these with the results obtained for the Trp mutants seems to indicate that W114 is the residue that would be most shielded by Chol. Not only that but its presence would also be required to establish the interactions with SM and Chol since its removal indicates that W110 is much more exposed to PC lipids. Since Trp residues have been shown to be unable to specifically interact with Chol by themselves^64^, Chol recognition would require W114 together with other residues.

This preferential interaction with Chol is not observed, in terms of quencher-shielding, for StnI. Since StnI also has an identical Trp residue (W115), the difference with StnII must lie somewhere else. Of the twelve residues that vary between StnI and StnII, only two are on the loops that directly interact with the membrane: D76 and T77 of StnII, and S77 and S78 of StnI. These residues are on the opposite loop to W110 and W114 (see Fig. 1). Depending on the exact nature of the interaction, oligomerization might be required if the mentioned residues all take part in the selectivity for Chol. This possibility cannot be ruled out, given that, according to our crosslinking analysis, StnII-I6C/K67C^ox^ also forms oligomers. The acidic character of the Asp residue in contrast with the polar character of Ser might tune these interactions, modulating what the lipid population is in the first shell of annular lipids of these pores.

Depth-dependent quenching did not report significant differences, though all proteins seem to have their Trp residues closer to the membrane midplane in membranes without Chol. It could be argued that this might be an effect of Chol on the depth of the quencher groups. However, Trp depth is measured relative to these quenchers. Hence, while the measurement may not be precise in terms of absolute distance to the bilayer center (in Å) because the actual depths of the quenchers might not the same as those measured in POPC^78^, these results do show that Trp residues of Stns are at a deeper position in the membrane relative to the acyl chains of the modified PC molecules. As expected, our results for the Trp mutants of StnII indicate that the average position of W110 is deeper than that of W114.

**Figure 5:**
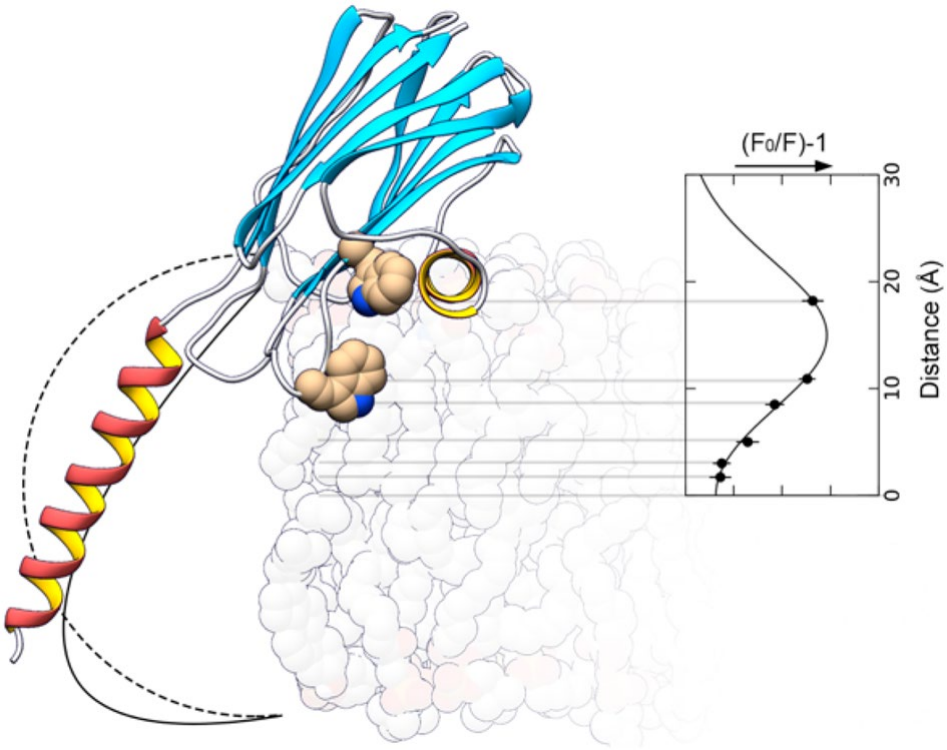
Position of the Trp residues of Stns within the membrane. The structure of StnI (PDB ID 2KS4), modified to present the N-terminal α-helix extended, showing W111 and W115 (W110 and W114 in StnII) in their expected position relative to the membrane normal, according to present results (insert on the right). The angle with the α-helix with the membrane normal is ~ 32º, according to^29,86^. The dashed line indicates the membrane shape in an ideal torus. Solid line is the suggestion of what the shape of the membrane limit (not necessarily the polar surface) could be in these pores. It seems unlikely that lipid headgroups could reside on the part of the line that is behind the α-helix. To see this figure in color, go online.

The good fits obtained in most cases also indicate that, if Stn pores are toroidal, lipid acyl-chains are still mostly parallel to the membrane normal in the region surrounding the Trp residues (Fig. 5). One of the goals of this analysis was to detect membrane curvature induced by pore formation if this in fact occurred. The results obtained do not give many hints in this regard. Nevertheless, together with past results on the angle of the N-terminal α-helix^29,93^, these new data do suggest that the outer leaflet of the vesicles, the one on which binding occurs, is unlikely to be curved (Fig. 5).

## Conclusions

Taken together, our results show that, regardless of their specific recognition of SM, Stn pores are also in contact with PC lipids. However, for StnII, but not for StnI, these contacts are reduced when Chol is present in the membrane. We propose that this preference, in which W114 may intervene, could perhaps be related to the difference between these toxins that occur in one of the loops that they insert in the membrane. Finally, it appears that acyl-chain orientation is not significantly altered by Stns, at least in the zone surrounding the membrane-embedded Trp residues of these proteins.

## Supplementary Information

Supplementary Information.

## Data Availability

The data supporting the findings of this study are available from the corresponding author (juan.palaciosb1a@gmail.com) upon reasonable request.

## Abbreviations

Chol: Cholesterol
CTL: 5,7,9,(11)-Cholestatrien-3β-ol
Cys: Cysteine
DMSO: Dimethyl sulfoxide
DSS: Disuccinimidyl suberate
DSS/P: DSS/protein
EqtII: Equinatoxin II
FWHM: Full width at half maximum
LUV: Large unilamellar vesicle(s)
L/P: Lipid/protein
PC: Phosphatidylcholine
SLPC: Spin-labeled PC
SM: Sphingomyelin
Stn: Sticholysin
T_m_: Melting temperature
Trp: Tryptophan
