# Author Correction: Determination of the boundary lipids of sticholysins using tryptophan quenching

**DOI:** 10.1038/s41598-023-36403-x

**Published:** 2023-06-08

**Authors:** Juan Palacios‑Ortega, Rafael Amigot‑Sánchez, Carmen García‑Montoya, Ana Gorše, Diego Heras‑Márquez, Sara García‑Linares, Álvaro Martínez‑del‑Pozo, J. Peter Slotte

**Affiliations:** 1 Biochemistry Department, Faculty of Science and Engineering, Åbo Akademi University, Turku, Finland; 2 Departamento de Bioquímica y Biología Molecular, Universidad Complutense, Madrid, Spain

Correction to: *Scientific Reports* 10.1038/s41598-022-21750-y, published online 15 October 2022

The Acknowledgements section in the original version of this Article was incomplete.

“This research was supported by the Juselius Foundation (J.P.S. and J.P.-O.) and by UCM-Banco Santander Grants PR75/18-21561, PR87/19-22556, and PR108/20-26896 (to A.M.-d.-P.).”

now reads:

“This research was supported by the Juselius Foundation (J.P.S. and J.P.-O.) and by UCM-Banco Santander Grants PR75/18-21561, PR87/19-22556, PR108/20-26896 and a REACT-EU grant from the Comunidad de Madrid to the ANTICIPA prorogued project of Complutense University of Madrid (to A.M.-d.-P.).”

The original Article has been corrected.

