# Supplementary material for: Determination of the boundary lipids of sticholysins using tryptophan quenching
Source: Sci Rep. 2022 Oct 15;12:17328. doi: 10.1038/s41598-022-21750-y (PMC9569322; doi:10.1038/s41598-022-21750-y)
Supplement: Supplementary file 1 — Supplementary Information. [file 41598_2022_21750_MOESM1_ESM.pdf]

Supporting Material for

Determination of the Boundary Lipids of Sticholysins using Tryptophan Quenching

Juan Palacios-Ortega,[1,2]* Rafael Amigot-Sánchez,[2] Carmen García-Montoya,[2] Ana Gorše,[2] Diego Heras-Márquez,[2] Sara García-Linares,[2] Álvaro Martínez-del-Pozo,[2] & J. Peter Slotte[1]

[1] Biochemistry, Faculty of Science and Engineering, Åbo Akademi University, Turku, Finland

[2] Departamento de Bioquímica y Biología Molecular, Universidad Complutense, Madrid, Spain

Corresponding author: Juan Palacios-Ortega
Email: juan.palaciosb1a@gmail.com (ORCID 0000-0002-4629-0221)

This file includes:

Supplemental methods and figures S1 to S11 + raw images of the gels shown in Fig.2 and Fig. S3.

Methods

Time-resolved data analysis

Intensity decays were analyzed using FluoFit Pro software from PicoQuant. The intensity decay curves ($I(t')$) were fit using a model consisting of the sum of discrete exponential terms [1]:

$$I(t') = \int_{-\infty}^{t} IRF(t') \sum_{i=1}^{n} \alpha_i \exp\left(-\frac{t - t'}{\tau_i}\right) dt', \qquad \text{eq.S1}$$

where α_i is the normalized amplitude of the ith component of the intensity decay characterized by a lifetime τ_i. The number of exponential terms was chosen as the smallest required to obtain a satisfactory fit. Judging from the value of the reduced χ^2, the distribution of the residuals and the trace of the autocorrelation plot, intensity decays of Stns' Trp emission can be fitted using three-component exponentials.

Analysis of the emission spectra of tryptophan

The parameters of the Trp emission spectra can be obtained by fitting the measured wavelength(λ)-dependent fluorescence signal with a log-normal distribution [2-5], as

$$F(\lambda) = \begin{cases} 0\ , & \lambda \leq \lambda_{max} - \frac{\rho\Gamma}{\rho^2-1} \\ F_0 \exp\left[\frac{-\ln 2}{\ln^2 \rho} \ln^2\left(1 + \frac{(\lambda-\lambda_{max})(\rho^2-1)}{\rho\Gamma}\right)\right], & \lambda > \lambda_{max} - \frac{\rho\Gamma}{\rho^2-1} \end{cases}, \qquad \text{eq.S2}$$

where λ_{max} is the wavelength at which the signal is maximum, F_0 is the intensity at λ_{max}, Γ is the width of the distribution at $F = F_0/2$ (full width at half maximum, or FWHM), and ρ describes the asymmetry of the distribution.

Analysis of tryptophan exposure to lipids

Analysis was performed using eq. 3. For the analysis, $F([Q])$ was normalized as F/F_0. Hence, the parameter F_0 in the equation had a value of 1. F_{min} was left as a floating parameter during the analysis since the emission of Stns on membranes composed entirely of quencher could not be measured because these toxins do not bind bilayers composed solely of PC. For these experiments, 7-SLPC was used since, *a priori*, this lipid was expected to quench both membrane-embedded sticholysin Trp residues (W110 and W114, StnII numbering).

This model relays on the assumption that observed quenching arises, essentially, from static processes [6]. To evaluate the nature of the quenching process, the emission of all samples was measured using both steady-state and lifetime measurements. Dynamic and static quenching are known to affect the steady-state signal [1]. However, the lifetimes retrieved from intensity decays are only affected by dynamic quenching and *not* by static quenching. Furthermore, the emission of selected samples was also measured while increasing the temperature. Dynamic quenching is known to increase with temperature, whereas static quenching is temperature independent, and may even decrease due to complex dissociation [1].

Effect of spin-labeled lipids on membrane binding by sticholysins

The change in fluorescence emission upon lipid addition was recorded as a function of time for all experiments in which $[L_T]$ was added in one step. This was to evaluate the effect of spin-labeled lipids on membrane binding kinetics of Stns. Integration time was 1 s. The Fluorimeter configuration was the same as above. The emission was recorded at 330 nm. Kinetic fluorescence traces were analyzed using

$$F(t) = \begin{cases} F(t)_\infty - \Delta F(t), & t < t_0 \\ F(t)_\infty - \Delta F(t)\ \sum_i^n \alpha_i \exp\left(-\frac{t - t_0}{\tau_i}\right), & t \geq t_0 \end{cases}, \qquad \text{eq.S3}$$

where $F(t)_\infty - \Delta F(t) = F(t)_0$, the initial fluorescence signal, $F(t)_\infty$ is the final fluorescent signal of the sample, $\Delta F(t)$ is the change in the signal ($\Delta F(t) = F(t)_\infty - F(t)_0$), t_0 is the time of lipid addition, and α_i and τ_i (in s) are the amplitudes and the corresponding characteristic times of change in the fluorescent signal, which, since a fixed wavelength was used, should be proportional to the rate of membrane binding [2]. Two exponential components were required to adequately fit the model to the data.

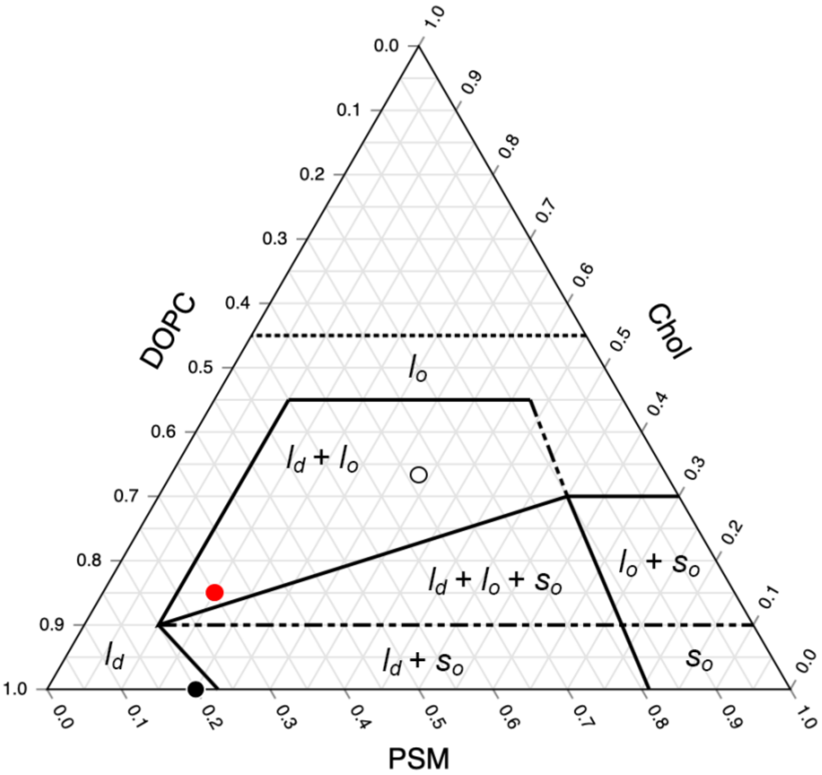

Figure S1. Phase diagram of the DOPC:PSM:Chol system at 23 °C, as determined by Nyholm *et al.* [7]. Solid lines represent boundaries determined by the authors of the paper [7], whereas dashed lines are theoretical and not revealed by tPA lifetimes. The dotted line represents the fraction of Chol at which the sterol is no longer incorporated in the membrane. The DOPC:PSM:Chol 1:1:1 composition is highlighted with an open circle, DOPC:PSM:Chol 70:15:15 is indicated with a solid red circle, and DOPC:PSM 80:20 is denoted with a solid black circle.

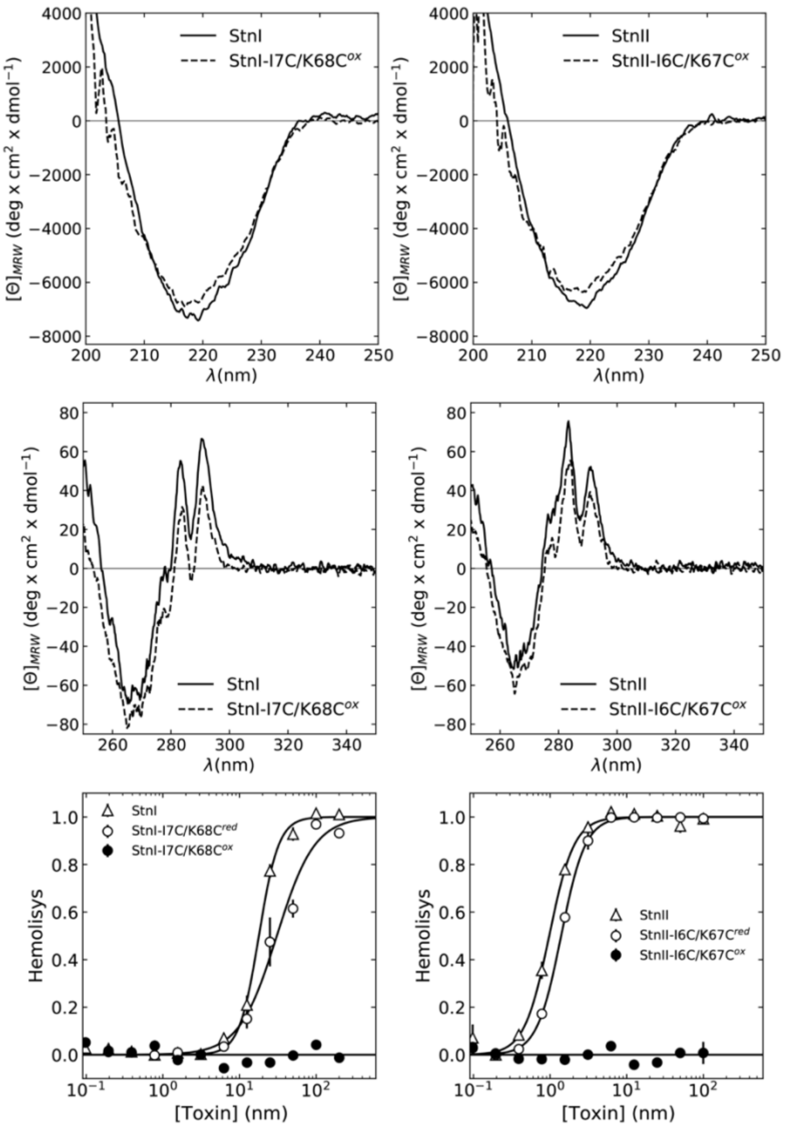

Figure S2. **Structural and functional characterization of the selectively active mutants of StnI and StnII.** Top panels show the far-UV CD spectra of both mutants (dashed lines) as compared to the spectra of the WT toxins (solid lines). The near-UV CD spectra, represented as above, are shown in the middle panels. Bottom row panels show the hemolysis results for the WT proteins (open triangles), the oxidized and inactive versions of the mutants (closed circles), and the reduced and reactivated versions of the mutants (open circles). Note how the activity is almost completely recovered upon disulfide reduction with DTT.

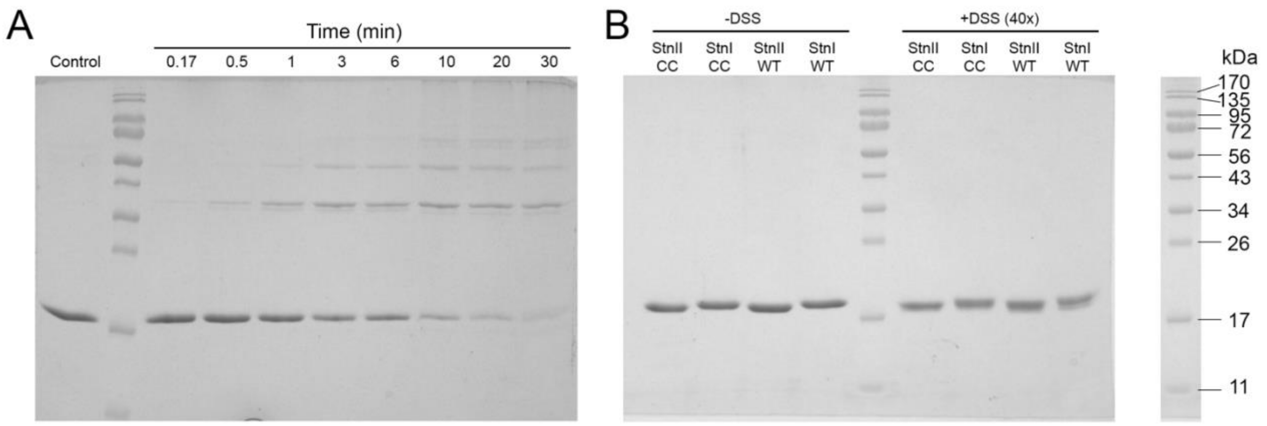

Figure S3. Optimization of the crosslinking experiments. A) Time scans with StnII-WT as control. StnII was incubated with DOPC:PSM:Chol 1:1:1 LUVs at a L/P ratio of 180 followed by DSS addition. The reaction was stopped with Gly at the reaction times indicated above. For the control, Gly was added before DSS. **B) Control experiments performed in absence of membranes.** The WT and inactive variants (indicated as WT and CC, for the double Cys mutation) of StnI and StnII were incubated with DSS for 10 min in absence of membranes. Results show that their electrophoretic mobility is essentially unchanged by DSS. However, observed bands are slightly broader. In fact, two bands can be distinguished for StnI-WT. This broadening probably reflects adducts formed by intramolecular crosslinking that slightly modify the molecular shape of the denatured protein. M_w of the standard proteins in the ladder are indicated on the right. The example image of the ladder is that shown in B.

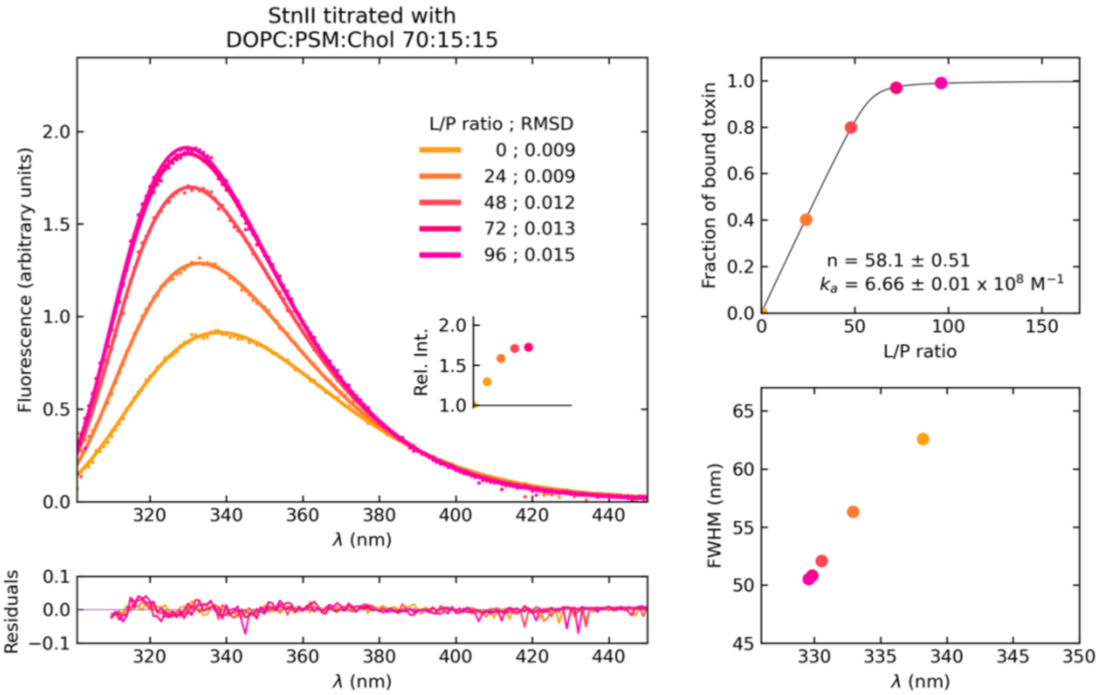

Figure S4. Titration of StnII with LUVs composed of DOPC:PSM:Chol in a molar ratio of 70:15:15. The main panel shows the measured Trp emission spectra recorded as explained in the Methods section. The inset shows the relative intensity of each spectrum. The spectra were fitted using eq. S2. Fitting residuals are shown below. Top right panel shows the fit of eq. 1 to the normalized intensity values, yielding the estimates for n and K_a shown in Table 1. Bottom right panel shows the λ of the emission maxima and the FWHM resulting from the fitting of eq. S2 to each spectrum. These calculations were made for all titration experiments.

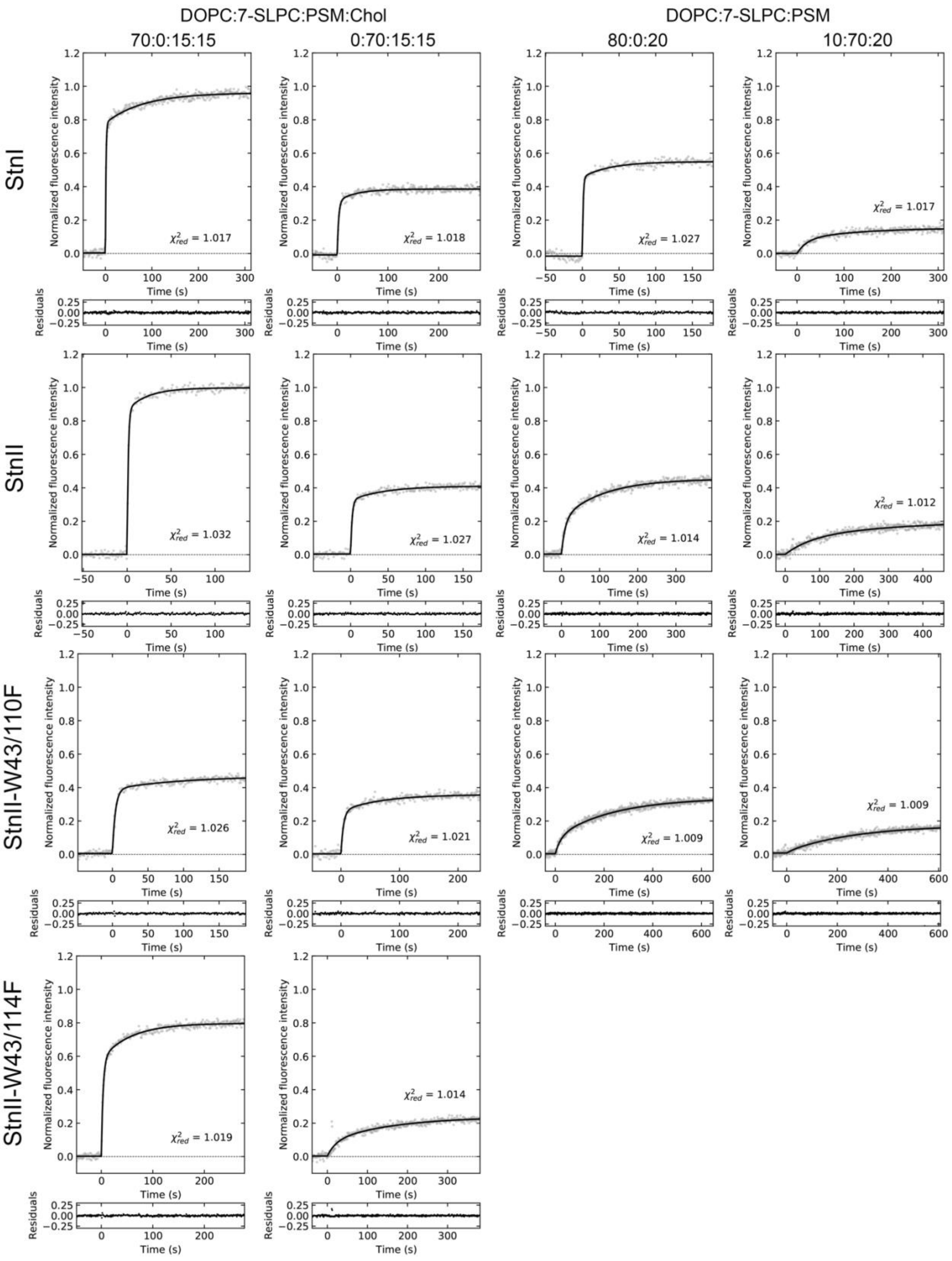

Figure S5. Membrane binding kinetics of Stns from Trp emission. Representative traces of the binding kinetics reported by Trp emission of StnI, StnII, StnII-W43/110F, and StnII-W43/114F to membranes with and without Chol, and with 0 mol% and 70 mol% of 7-SLPC. Solid lines are fits of eq. S3, which were used to obtain binding times. Similar traces were obtained for the inactive double Cys mutants, not shown. The scale was normalized to allow better comparison between traces.

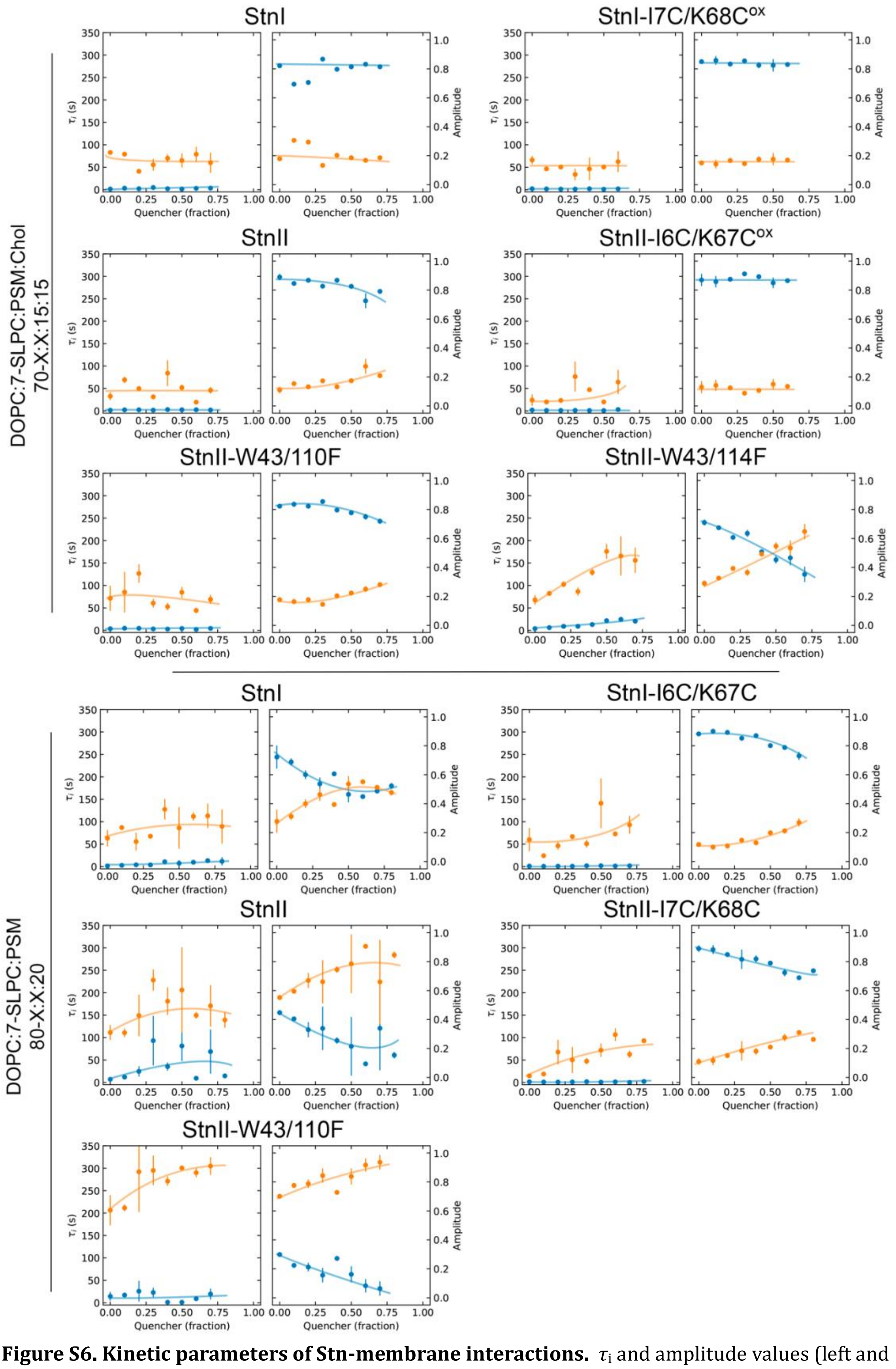

Figure S6. Kinetic parameters of Stn-membrane interactions. τ_i and amplitude values (left and right panels of each pair, respectively) were obtained from fitting eq. 4 to the time-dependent Trp emission traces. Colors indicate the correspondence of amplitude and τ_i each component resulting from fitting (one color each). Note that increasing quencher concentration has a larger effect on Stn

binding when Chol is absent from the membranes. Data are average ± SEM of n = 2-3. Solid lines are guides to the eye.

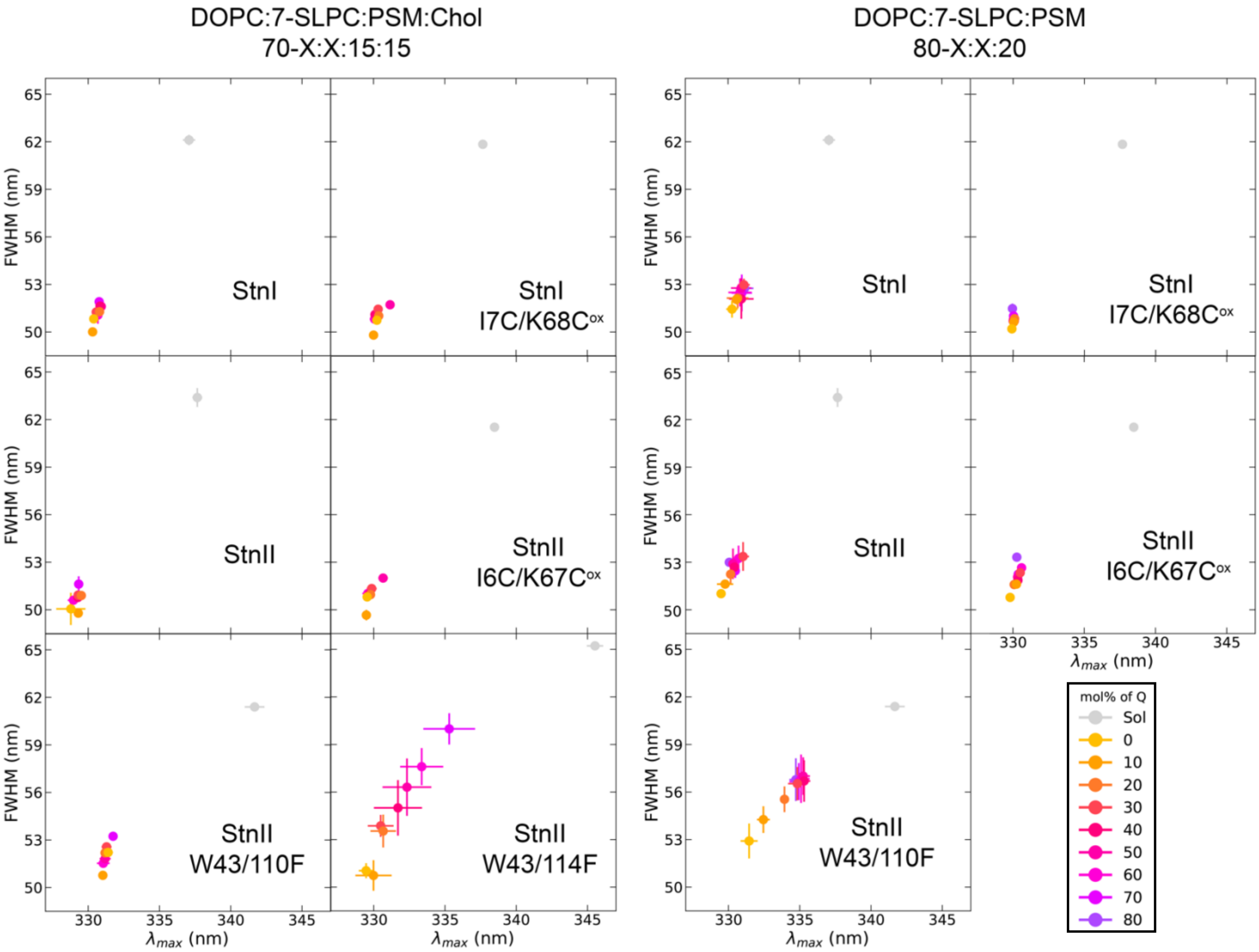

Figure S7. Effect of 7-SLPC on the FWHM and λ$_{max}$ of the emission spectra of the studied Stn variants. Plots FWHM as a function of λ$_{max}$ obtained from fitting eq. S2 to the emission spectra of the indicated Stn variant when bound to vesicles. Symbol color indicates mol% of 7-SLPC in the membrane. The values for the toxin in solution are indicated in gray. The FWHM and λ$_{max}$ of the spectra of each protein are also shown in grey. Values are average ± SEM of n = 2-3.

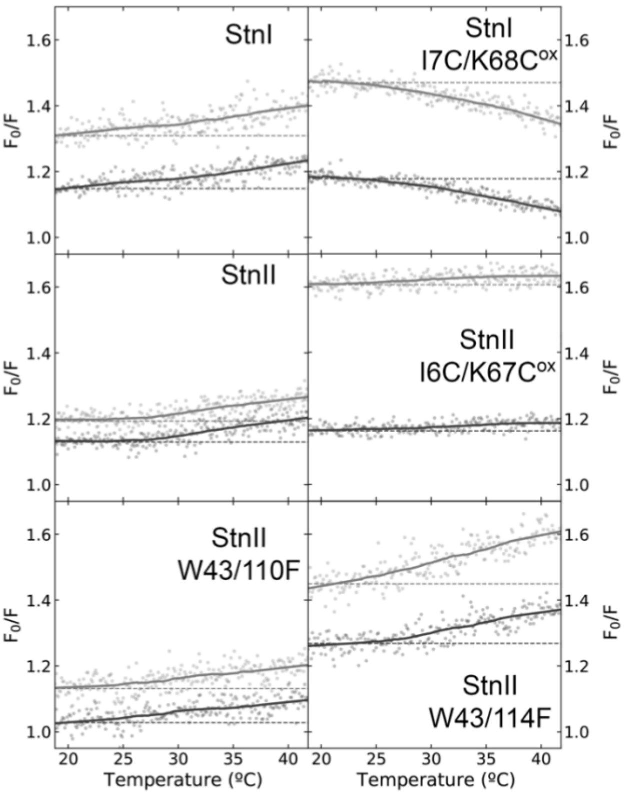

Figure S8. Temperature-dependent quenching in Chol-containing membranes. Steady-state F_0/F traces were obtained for the emission of the indicated Stn variants in membranes composed of DOPC:7-SLPC:PSM:Chol in molar ratios of 40:30:15:15 (black) and 0:70:15:15 (light gray). Solid lines are the smoothed trace of the raw data (shown as points). Dashed lines indicate the average value of the raw data at the beginning of each trace so that changes at higher temperatures are more easily appreciated.

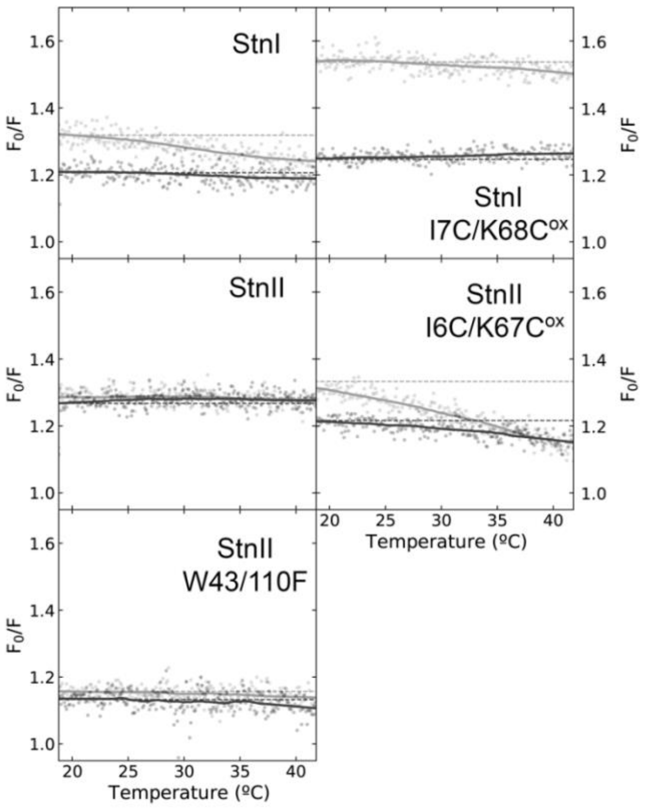

Figure S9. Temperature-dependent quenching in membranes without Chol. Steady-state F_0/F traces were obtained for the emission of the indicated Stn variants in membranes composed of DOPC:7-SLPC:PSM in molar ratios of 50:30:20 (black) and 10:70:20 (light grey). Solid lines are the smoothed trace of the raw data (shown as points). Dashed lines indicate the average value of the raw data at the beginning of each trace so that changes at higher temperatures are more easily appreciated.

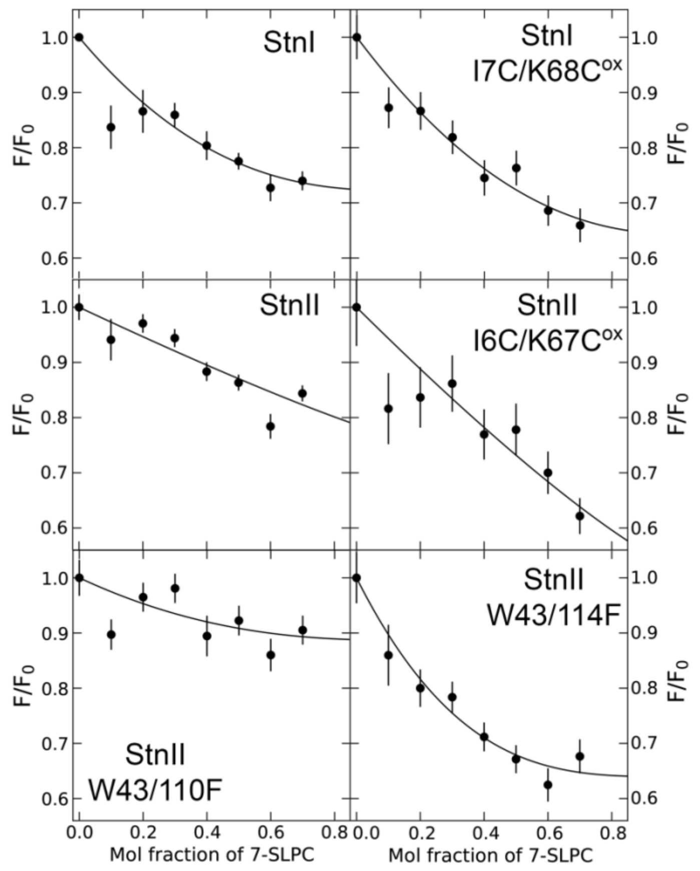

Figure S10. Fits of eq.2 to obtain the exposure and number of accessible lipids in membranes with Chol. Plots of F/F_0 for all toxins in membranes composed of DOPC:7-SLPC:PSM:Chol 70-X:X:15:15. The F/F_0 value projected for membranes composed solely of 7-SLPC is related to the fraction of emission shielded from the quencher. The number of PC lipids in contact with accessible Trp residues is related to the shape of the curve.

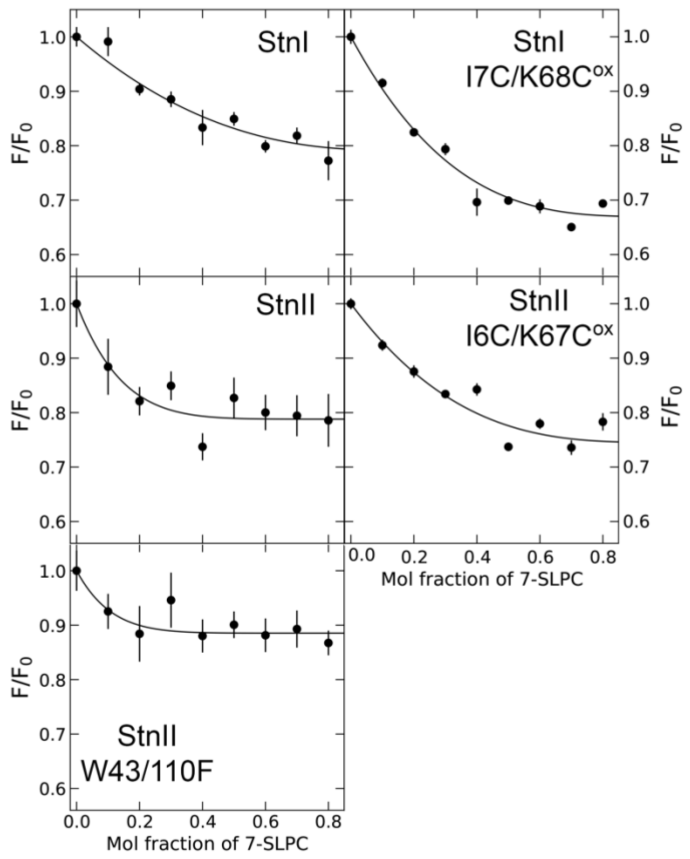

Figure S11. Fits of eq.2 to obtain the exposure and number of accessible lipids in membranes without Chol. Plots of F/F_0 for all toxins in membranes composed of DOPC:7-SLPC:PSM:Chol 70-X:X:15:15. AS before, the F/F_0 value projected for membranes composed solely of 7-SLPC is related to the fraction of emission shielded from the quencher. The number of PC lipids in contact with accessible Trp residues is related to the shape of the curve.

REFERENCES

1. Lakowicz, J.R. 2006. **Principles of fluorescence spectroscopy**, Springer Science & Business Media, ISBN: 1475730616.
2. Ladokhin, A.S., Jayasinghe, S., White, S.H. (2000). **How to measure and analyze tryptophan fluorescence in membranes properly, and why bother?** *Anal biochem*, 285 (2), 235-245.
3. Ladokhin, A.S., Wang, L., Steggles, A., Holloway, P.W. (1991). **Fluorescence study of a mutant cytochrome b5 with a single tryptophan in the membrane-binding domain**. *Biochemistry*, 30 (42), 10200-10206.
4. Burstein, E.A., Emelyanenko, V.I. (1996). **Log‐normal description of fluorescence spectra of organic fluorophores**. *Photochem Photobiol*, 64 (2), 316-320.
5. Burstein, E.A., Abornev, S.M., Reshetnyak, Y.K. (2001). **Decomposition of protein tryptophan fluorescence spectra into log-normal components. I. Decomposition algorithms**. *Biophys J*, 81 (3), 1699-1709.
6. London, E., Feigenson, G.W. (1981). **Fluorescence quenching in model membranes. 1. Characterization of quenching caused by a spin-labeled phospholipid**. *Biochemistry*, 20 (7), 1932-1938.
7. Nyholm, T.K., Lindroos, D., Westerlund, B., Slotte, J.P. (2011). **Construction of a DOPC/PSM/cholesterol phase diagram based on the fluorescence properties of trans-parinaric acid**. *Langmuir*, 27 (13), 8339-8350.

Fig. 2 Raw gel

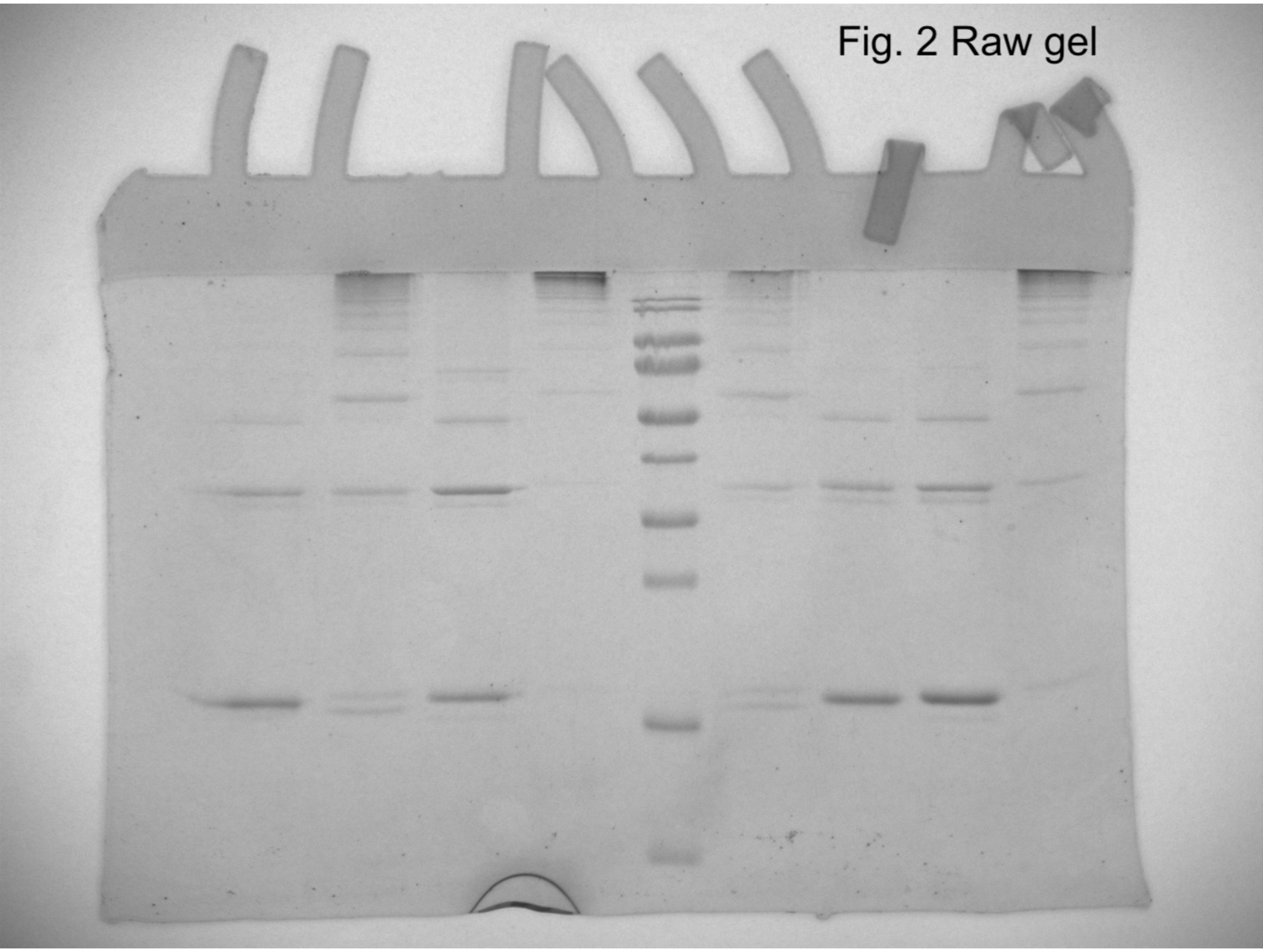

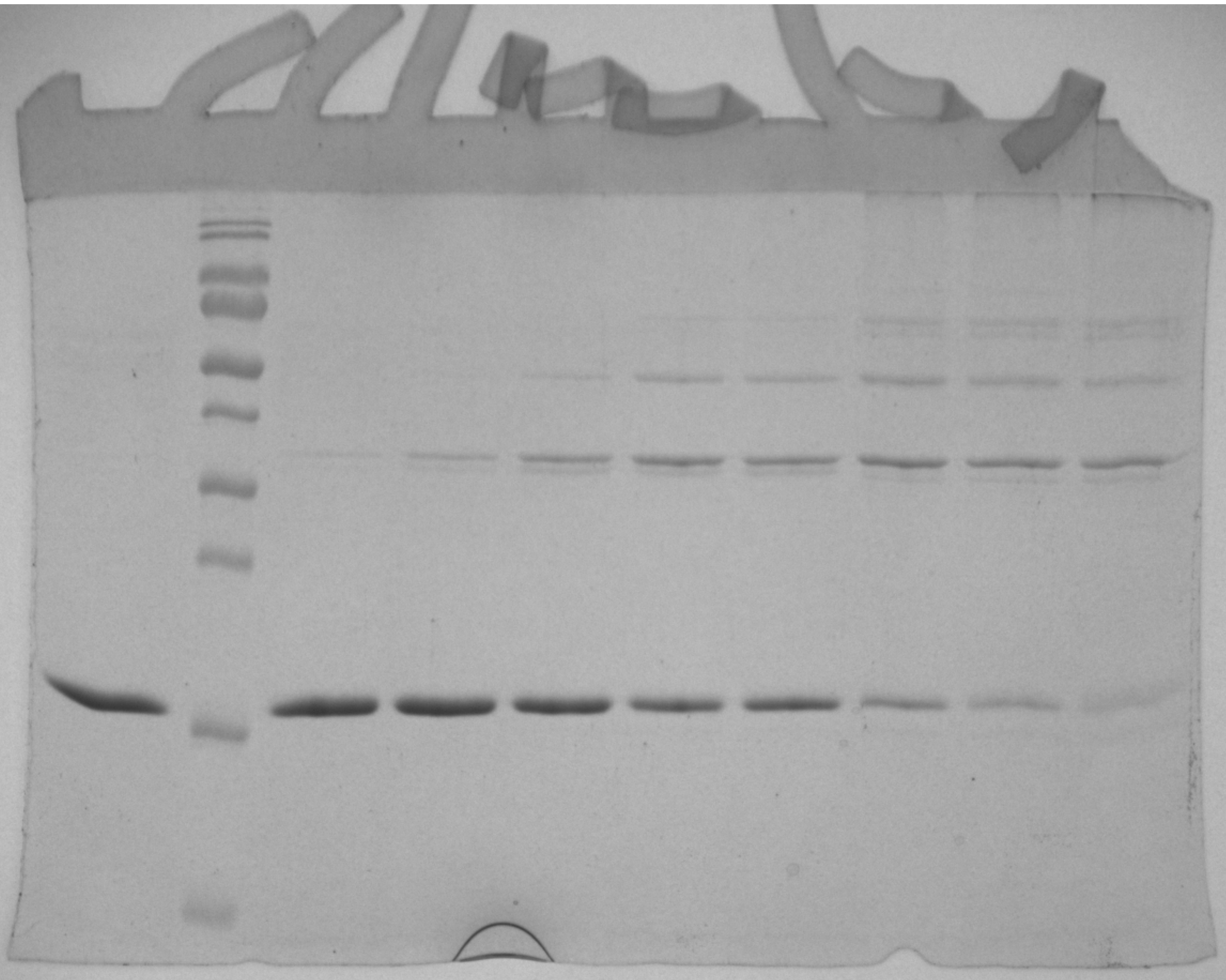

Fig. S3A Raw gel

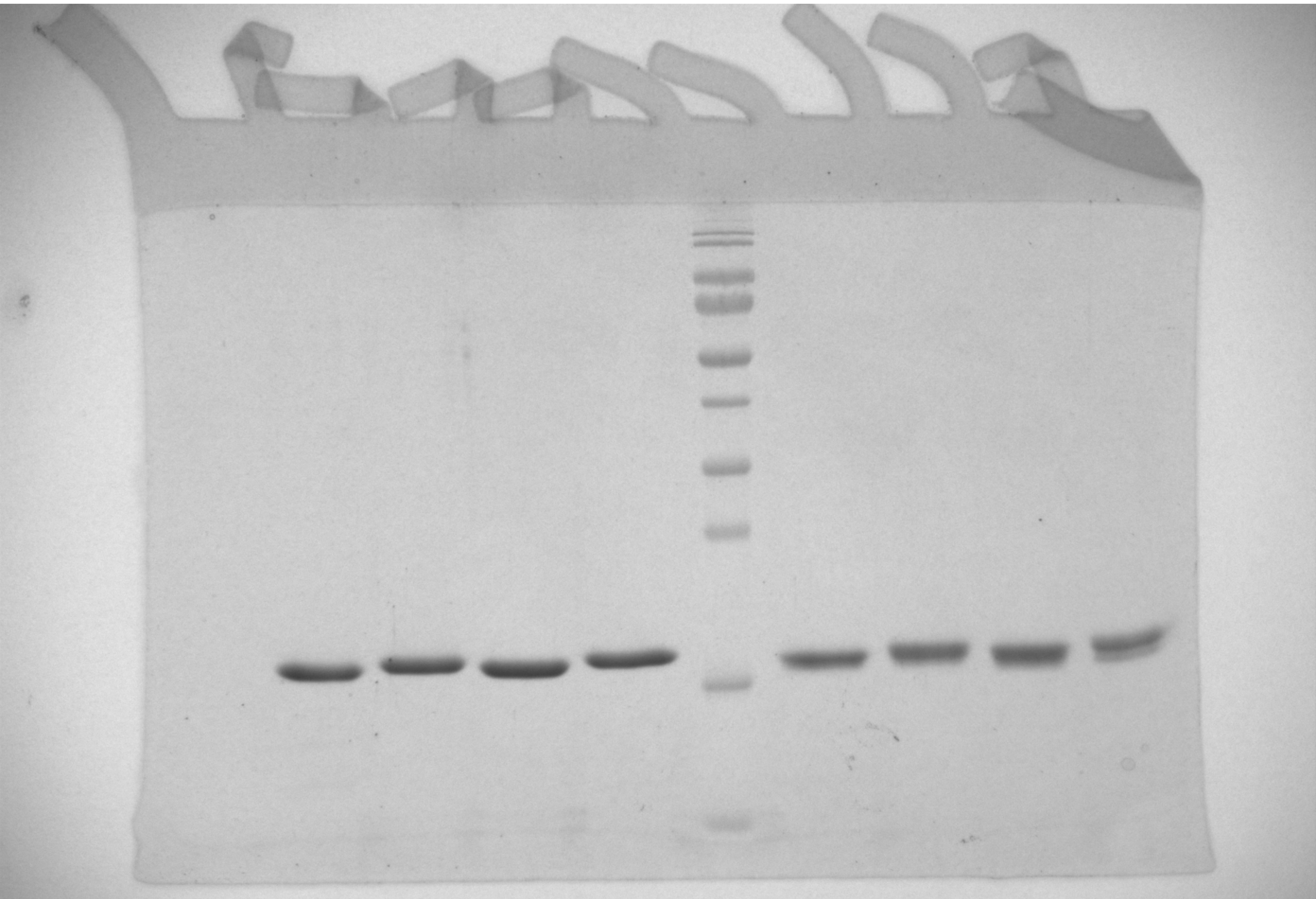

Fig. S3B Raw gel
